# Surgical Versus Non-Surgical Treatment for Vertebral Compression Fracture with Osteopenia: A Systematic Review and Meta-Analysis

**DOI:** 10.1371/journal.pone.0127145

**Published:** 2015-05-28

**Authors:** Jia-Bao Guo, Yi Zhu, Bing-Lin Chen, Bin Xie, Wen-Yi Zhang, Yu-Jie Yang, Yu-Shan Yue, Xue-Qiang Wang

**Affiliations:** 1 Second School of Clinical Medical, Nanjing University of Chinese Medicine, Nanjing, Jiangsu, China; 2 Department of sport Rehabilitation, Shanghai University of Sport, Shanghai, China; Toronto Western Hospital, CANADA

## Abstract

**Background:**

Surgical and non-surgical interventions are the two categories for treatment of vertebral compression fractures (VCFs). However, there is clinical uncertainty over optimal management. This study aimed to examine the safety and effectiveness of surgical management for treatment of VCFs with osteopenia compared with non-surgical treatment.

**Methods:**

We conducted a systematic search through electronic databases from inception to June 2014, with no limits on study data or language. Randomized controlled trials (RCTs) evaluating surgical versus non-surgical interventions for treatment of patients with VCFs due to osteopenia were considered. Primary outcomes were pain and adverse effects. A random-effects model was used to calculate the pooled mean difference (MD) or risk ratios with 95% confidence interval (CI).

**Results:**

Sixteen reports (11 studies) met the inclusion criteria, and provided data for the meta-analysis with a total of 1,401 participants. Compared with conservative treatment, surgical treatment was more effective in reducing pain (short-term: MD -2.05, 95% CI -3.55 to -0.56, P=0.007; mid-term: MD -1.70, 95% CI -2.78 to -0.62, P=0.002; long-term: MD -1.24, 95% CI -2.20 to -0.29, P=0.01) and disability on the Roland–Morris Disability score (short-term: MD -4.97, 95% CI -8.71 to -1.23, P=0.009), as well as improving quality of life on the Short-Form 36 Physical Component Summary score (short-term: MD 5.53, 95% CI 1.45 to 9.61, P=0.008) and the Quality of Life Questionnaire of the European Foundation for Osteoporosis score (short-term: MD -5.01, 95% CI -8.11 to -1.91, P=0.002). Indirect comparisons between vertebroplasty and kyphoplasty found no evidence that the treatment effect differed across the two interventions for any outcomes assessed. Compared with the sham procedure, surgical treatment showed no evidence of improvement in pain relief and physical function. Based on these two comparisons, no significant difference between groups was noted in the pooled results for adverse events.

**Conclusion:**

Compared to conservative treatment, surgical treatment was more effective in decreasing pain in the short,mid and long terms. However, no significant mid- and long-term differences in physical function and quality of life was observed. Little good evidence is available for surgical treatment compared with that for sham procedure. PV and BK are currently used to treat VCFs with osteopenia, with little difference in treatment effects. Evidence of better quality and from a larger sample size is required before a recommendation can be made.

**Systematic Review Registration:**

http://www.crd.york.ac.uk/PROSPERO PROSPERO registration number: CRD42013005142.

## Introduction

An estimated 1.4 million vertebral compression fractures (VCFs) are reported worldwide every year [1]. Patients with VCF may present with severe back pain, functional disability, and a decrease in quality of life [2, 3]. The most common etiology of VCFs is osteoporosis, although trauma, infection, and tumour can also lead to VCFs. Bone metastases and multiple myeloma can lead to generalised osteoporosis or weakening of bone at specific sites [4]. As a result, fractures were caused, especially painful VCFs. Aging is associated with a decrease in bone mineral density [5–7]. VCFs secondary to osteopenia arising from osteoporosis, fall or tumours are a cause of morbidity in older adults. These fractures will increase with the increasing aging of the population [8, 9].

Surgical and non-surgical methods are used to treat VCFs. Standard non-surgical treatment includes bed rest, analgesia, use of a back brace or corset, and physical support. Minimally invasive techniques, such as percutaneous vertebroplasty (PV) and balloon kyphoplasty (BK), are popular for treating painful VCFs [10–13]. PV involves the percutaneous injection of bone cement into the affected vertebra to stabilize the fractured vertebral body, and results in immediate pain relief. It was initially devised in France by Deramond and Galibert in 1984, and first performed to treat hemangioma in the cervical spine [14, 15]. Since then, the operation has become widely used and proved valuable in treating osteoporotic VCFs. BK is a type of PV in which inflatable bone tamps are used to restore vertebral height. After removing the balloons, the resulting intravertebral cavity is filled with bone cement to stabilize the vertebral body. No consensus has been reached over which is the best management strategy for patients with VCFs.

Several published systematic reviews have assessed the effects of surgical management for the treatment of VCFs [16–19]. However, previous reviews focused on one type of surgical management, such as PV or BK. To synthesise the latest trial reports, we have performed this systematic review and meta-analysis of randomized controlled trials (RCTs) of surgical management for treatment of VCFs with osteopenia compared with non-surgical treatment. This review includes trials evaluating a variety of different surgical methods as used to treat people with VCFs due to osteopenia, which provide an overall assessment on the use of surgical managements in this patients population. To our best knowledge, this study is the first comprehensive meta-analysis of surgical management versus non-surgical management in this issue.

## Methods

### Search Strategy

We did a systematic review in accordance with the PRISMA (Preferred Reporting Items for Systematic Reviews and Meta-Analyses) guidelines [20]. For a completed PRISMA checklist, see S1 Checklist. The Cochrane Central Register of Controlled Trials, PubMed, EMBASE, Web of Science, China Biology Medicine disc, China National Knowledge Infrastructure, and Wanfang Database were searched from the earliest available date to June 2014. We used “Vertebroplasty”, “Fractures, Bone” and “RCTs” as search terms (full details of the search in S1 File). Reference lists of selected articles were reviewed for other potentially relevant citations.

### Inclusion Criteria

#### Types of studies

Only RCTs investigating surgical versus non-surgical interventions for the treatment of patients with VCFs were included. No limits on publication dates or any language restrictions were placed.

#### Types of participants

Patients with VCFs due to osteopenia arising from osteoporosis or tumours were included. We excluded patients who had severe cardiopulmonary comorbidity, untreatable coagulopathy, systemic or local spine infection, suspected underlying malignant disease, radicular syndrome, spinal cord compression syndrome, or contraindications to magnetic resonance imaging.

#### Types of interventions

We included an experimental group that received surgical treatment, and a control condition in which either conservative treatment or a sham procedure was administered. Surgical treatments included vertebroplasty, kyphoplasty, pedicle screw fixation, anterior reconstruction or fusion, and posterior reconstruction or fusion. Patients in the sham surgical intervention group underwent the same procedure as those in the experimental group up to the insertion of the needle. Conservative interventions consisted of bed rest, medication (e.g., analgesics), use of a corset, brace, or cast, and rehabilitation (e.g., physiotherapy).

#### Types of outcome measures

The primary outcomes were self-reported pain and adverse effects. All reported symptomatic and asymptomatic adverse events were recorded. Secondary outcomes were function or disability, health-related quality of life, and hospitalization costs. The costs included procedural costs, rehabilitation costs, and primary costs. All outcome measures were pre-specified before review commencement. We categorized outcomes as short-term (not longer than three months), mid-term (six months), and long-term (twelve months or more) follow-up. All outcome measures were pre-specified before review commencement.

### Data Extraction

Two reviewers (Guo JB and Xie B) independently extracted and cross-checked data on trials. The decision to include studies was made initially on the basis of the study title and abstract. When a study could not be excluded with certainty at this stage, the full-text was obtained for evaluation. Disagreements were resolved by discussion and, where necessary, in consultation with a third reviewer (Zhang WY). Extracted information included the first author, publication year, study design (e.g., intervention, follow-up length, outcome measures), characteristics of participants (e.g., age, gender, inclusion/exclusion criteria, sample size, duration of complaint), and information to assess the risk of bias.

### Quality Assessment

The risk of bias of the included studies was independently assessed by two authors (Guo JB and Chen BL) using the Cochrane Collaboration’s “Risk of Bias” [21]. We assessed the following domains: sequence generation, allocation concealment, blinding of participants and personnel, blinding of outcome assessment, incomplete outcome data, selective outcome reporting, and other sources of bias. Risk of bias was categorized as low, unclear, or high for each of the included studies. Disagreements were resolved by third party adjudication.

### Statistical Analysis

Quantitative data were entered into Review Manager (RevMan 5.1). When appropriate, results of comparable groups of studies were pooled in a meta-analysis using the random-effects model. For dichotomous outcomes, risk ratios (RRs) with 95% confidence intervals (CIs) were calculated. For continuous outcomes, mean difference (MDs) with 95% CIs or standardized mean differences with 95% CI were calculated. P values were 2-tailed, and a value of P<0.05 was considered statistically significant. Heterogeneity between studies was measured by Chi^2^ statistic (P<0.1) and quantified with I² statistic [22]. We judged the I^2^ values of less than 25% to be minimal heterogeneity, less than50% to be moderate heterogeneity, and 50% or greater to be substantial heterogeneity. In attempting to dissipate any heterogeneity, subgroup analyses were performed on studies. Since the different trials implemented various types of surgical management, trials were divided according to the type of intervention (e.g., PV, BK). If there were two or more surgical methods in the same comparison, then the subgroup analysis was conducted. Potential publication bias was evaluated using funnel plots.

When studies required for the meta-analysis did not provide means and standard deviations at follow-up, we contacted corresponding authors to obtain the unreported data. In the few cases in which a response was unavailable, we estimated the standard deviations using the formula suggested in the Cochrane Handbook for Systematic Reviews of Interventions [23].

## Results

**Fig 1:**
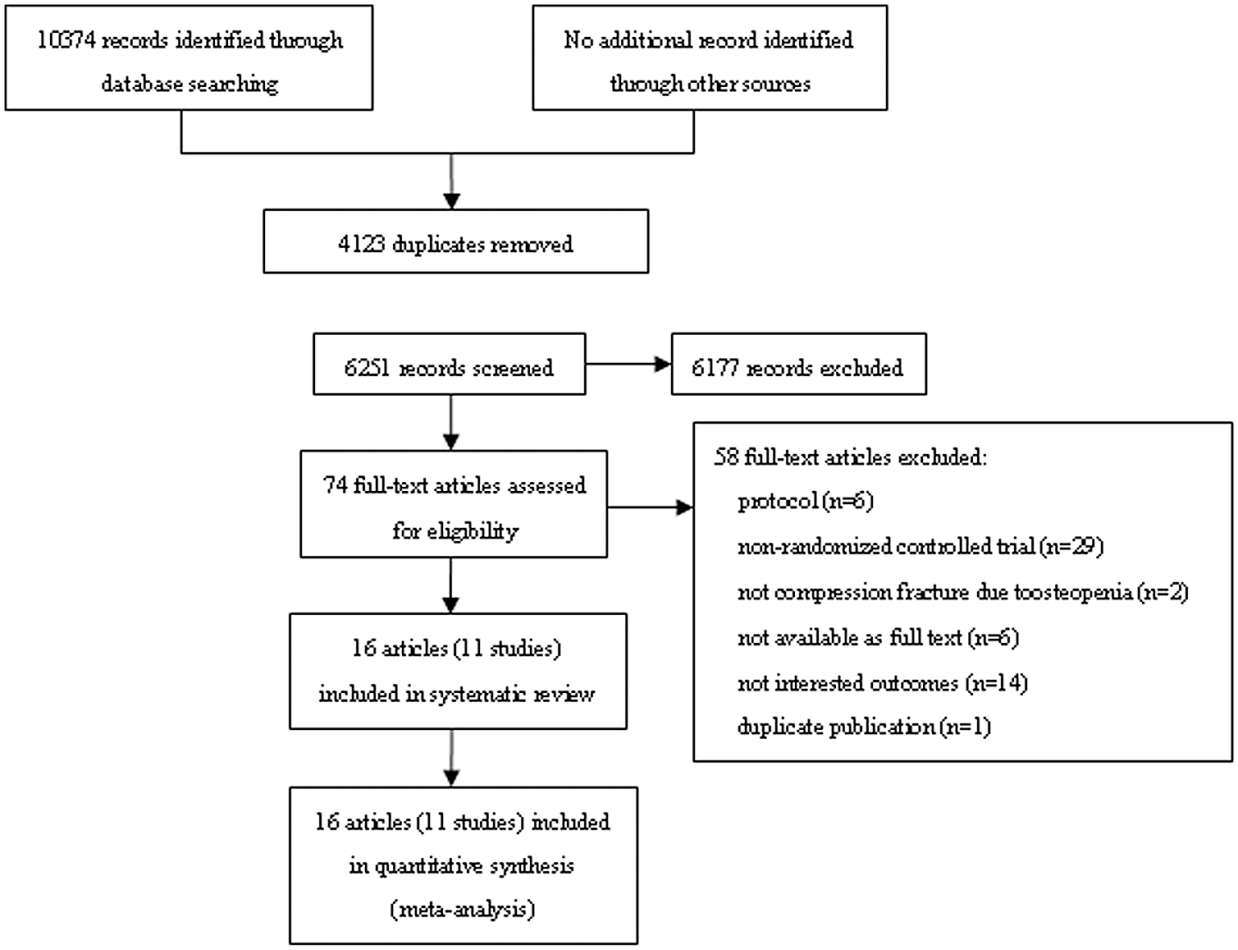
Flow chart of study selection. For details of study identification.

Our search identified a total of 10,374 records. After removing duplicates, 6,251 records were screened. A further 74 were determined to be potentially relevant for full-text review. Among these studies, 11 studies (16 reports) met the inclusion criteria [24–39], and provided data for the meta-analysis. A manual search of reference lists from these studies did not yield any new eligible study (Fig 1).

### Study characteristics

Eleven studies (16 reports) were included, and a summary of their characteristics is shown in Table 1. Six studies [27, 29, 34–36, 38, 39] were small single-center trials, and five [24, 25, 27, 28, 30–33, 37] were multicenter trials. All the studies used surgical treatment as the intervention. Two types of interventions were used; eight studies [24–26, 30–35, 38, 39] used PV, three studies [27–29, 36, 37] used BK. Regarding controls, ten studies [26–29, 31–39] used conservative treatment and two [24, 25, 30] used sham surgical treatment. All participants had VCFs due to osteopenia. One study [37] arised from bone metastases and multiple myeloma. Another study [27] was caused by either osteoporosis or tumours, and the other participants all had osteoporotic VCFs. Data from 1,401 participants were extracted, of whom 701 received surgical treatment.

**Table 1:** Characteristics of included studies.

Article	Year	Centers	Comparison arms	Sample Size, N	Duration of VCF	Mean (SD/Range) Age, y	Outcomes	Time point
Voormolen[26]	2007	The Netherland	G1: PV; G2: conservative treatment	G1: 18; G2: 16	G1: ≥ 6 wk and ≤ 6 mo; G2: ≥ 6 wk and ≤ 6 mo	G1: 72 (59–84); G2: 74 (55–88)	Pain (VAS 0–10); Adverse events Disability (RMD); Quality of life (QUALEFFO)	1 d; 2 wk
Wardlaw[27]	2009	21 in AUS, Belgium, France, Germany, Italy, the Netherlands, Sweden and UK	G1: BK; G2: conservative treatment	G1: 149; G2: 151	G1: ≥ 3 mo; G2: ≥ 3 mo	G1: 72.2 (9.3); G2: 74.1 (9.4)	Pain (0–10); Adverse events; Disability (RMD); Quality of life (EQ-5D, SF-36 PCS)	1mo;3 mo; 6 mo; 1 y
Boonen[28]	2011	21 in AUS, Belgium, France, Germany, Italy, the Netherlands, Sweden and UK	G1: BK; G2: conservative treatment	G1: 149; G2: 151	G1: ≥ 3 mo; G2: ≥ 3 mo	G1: 72.2 (9.3); G2: 74.1 (9.4)	Pain (0–10); Adverse events; Disability (RMD); Quality of life (EQ-5D, SF-36 PCS)	2 y
Fritzell[29]	2011	Swedish	G1: BK; G2: conservative treatment	G1: 35; G2: 32	G1: ≤ 3 mo; G2: ≤ 3 mo	G1: 72.2 (10.1); G2: 75 (9.7)	Hospitalization costs	1 mo; 3 mo; 6 mo; 1 y; 2 y
Buchbinder[24]	2009	4 in AU	G1: PV; G2: sham procedure	G1: 38; G2: 40	G1: < 6 wk or ≥ 6 wk; G2: < 6 wk or ≥ 6 wk	G1: 72.4 (14.0); G2: 78.9 (9.5)	Pain (0–10); Adverse events; Disability (RMD); Quality of life(QUALEFFO, EQ-5D)	1 wk; 1 mo; 3 mo; 6 mo
Kallmes[25]	2009	5 in US; 5 in UK; 1 in AU	G1: PV; G2: sham procedure	G1: 68; G2: 63	G1: ≥1 y; G2: ≥1 y	G1: 73.4 (9.4); G2: 74.3 (9.6)	Pain(0–10); Adverse events; Disability (RMD); Quality of life (SF-36 PCS, SF-36 MCS, EQ-5D)	3 d; 14 d; 1 mo; 3 mo
Comstock[30]	2013	5 in US; 5 in UK; 1 in AU	G1: PV; G2: sham procedure	G1: 68; G2: 63	G1: ≥1 y; G2: ≥1 y	G1: 73.4 (9.4); G2: 74.3 (9.6)	Pain(0–10); Disability (RMD)	6 mo; 1 y
Chen[31]	2010	China	G1: PV; G2: conservative treatment	G1: 18; G2: 22	G1:≤ 6 wk; G2:≤ 6 wk	G1: 77.5 (0.8); G2: 76.3 (0.5)	Pain (VAS 0–10); Adverse events	3 mo
Klazen[32]	2010a	5 in the Netherlands; 1 in Belgium	G1: PV; G2: conservative treatment	G1: 101; G2: 101	G1: ≤ 6 wk; G2: ≤ 6 wk	G1: 75.2 (9.8); G2: 75.4 (8.4)	Pain (VAS 0–10); Disability (RMD); Quality of life (QUALEFFO); Hospitalization costs	1 d; 1 wk; 1 mo; 3 mo; 6 mo; 1 y
Klazen[33]	2010b	5 in the Netherlands; 1 in Belgium	G1: PV; G2: conservative treatment	G1: 101; G2: 101	G1: ≤ 6 wk; G2: ≤ 6 wk	G1: 75.2 (9.8); G2: 75.4 (8.4)	Adverse events	1 y
Rousing[34]	2009	Denmark	G1: PV; G2: conservative treatment	G1: 25; G2: 24	G1: ≤ 8 wk; G2: ≤ 8 wk	G1: 80 (65–96); G2: 80 (71–93)	Pain (VAS 0–10); Quality of life (EQ-5D, SF-36 PCS, SF-36 MCS)	3 mo
Rousing[35]	2010	Denmark	G1: PV; G2: conservative treatment	G1: 25; G2: 24	G1: ≤ 8 wk; G2: ≤ 8 wk	G1: 80 (65–96); G2: 80 (71–93)	Pain (VAS 0–10); Adverse events; Quality of life (EQ-5D, SF-36 PCS, SF-36 MCS)	1 y
Xie[36]	2011	China	G1:BK; G2: conservative treatment	G1: 77; G2: 87	G1: mean (SD): 3.1 d (2.0); G2: mean (SD): 3.2 d (2.0)	G1: 67 (10); G2: 67 (7)	Pain (NRS 0–10); Adverse events; Quality of life (SF-36 PCS, SF-36 MCS)	1 d; 6 mo
Berenson[37]	2011	22 in Europe, the USA, Canada, and Australia	G1: BK; G2: conservative treatment	G1: 68; G2: 61	NA	G1: 64.8 (37.6–88.0); G2: 63.0 (39.5–83.4)	Pain (NRS 0–10); Adverse events; Disability (RMD)Quality of life (SF-36 PCS, SF-36 MCS)	1 wk; 1 mo; 3 mo; 6 mo; 1 y
Farrokhi[38]	2011	Iran	G1: PV; G2: conservative treatment	G1: 40; G2: 42	G1: ≥ 4 wk and ≤ 1 y; G2: ≥ 4 wk and ≤ 1 y	G1: 72 (59–90); G2: 74 (55–87)	Pain (NRS 0–10); Adverse events; Quality of life (ODI)	1 wk; 2 mo; 6 mo; 1 y; 2 y; 3 y
Blasco[39]	2012	Spain	G1: PV; G2: conservative treatment	G1: 64; G2: 61	G1: ≤ 1 y; G2: ≤ 1 y	G1: 71.33 (9.95); G2: 75.27 (8.53)	Pain (VAS 0–10); Adverse events; Quality of life (QUALEFFO)	2 wk; 2 mo; 6 mo; 1 y

**Abbreviations** G: Group; SD: Standard deviation; PV: percutaneous vertebroplasty; BK: balloon kyphoplasty; VCF: vertebral compression fracture; VAS: Visual Analog Scale; NRS: Numerical Rating Scale; RMD: Roland-Morris Disability; ODI: Oswestry Disability Index; SF-36 PCS: the Short-Form 36 Physical Component Summary; SF-36 MCS: the Short-Form 36 Mental Component Summary; EQ-5D: the European Quality of Life–5 Dimensions; QUALEFFO: the Quality of Life Questionnaire of the European Foundation for Osteoporosis; NA: not available.

### Qualitative Assessment

Methodological details are reported in S1 and S2 Figs. Three (18.75%) trials [31, 27–37, 39] were not provided with allocation concealment, which resulted in unclear risk of selection bias. Thirteen (81.25%) trials [26–29, 31–39] were at high risk of performance bias, reflecting the difficulty in blinding surgeons. Only one (6.25%) trial [36] did not describe the patients who withdrew from the study. Finally, 11 (68.75%) trials [24, 25, 27–30, 32–33, 37–39] stated per protocol the primary method of analysis, and the remaining trials did not describe the method of analysis.

## Comparison 1: Surgical Versus Conservative Interventions for Treating VCF with Osteopenia

### Self-related pain

**Fig 2:**
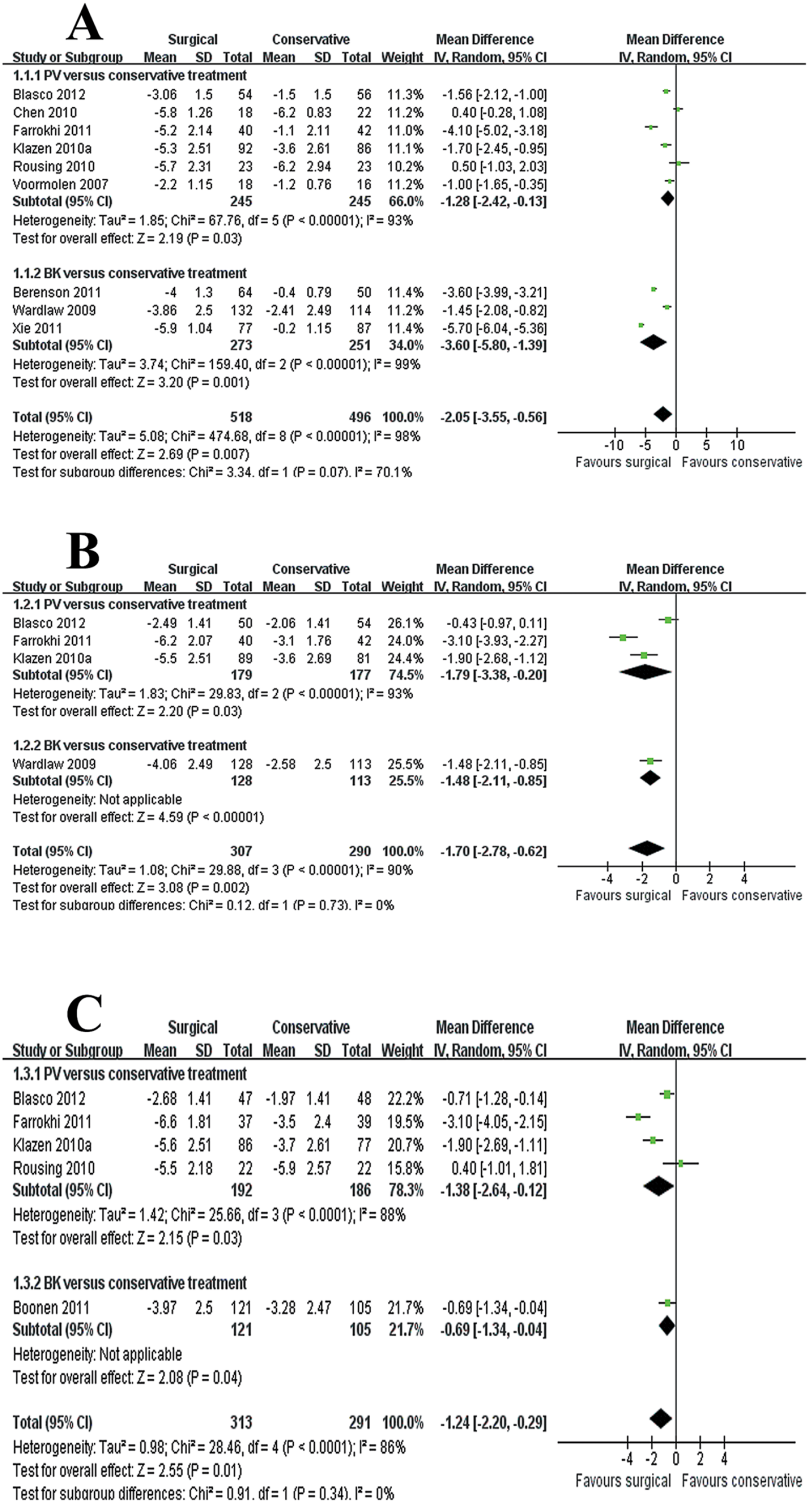
Self-related pain for surgical versus conservative treatment. A: mean difference (MD) at the end of the intervention (not longer than 3 months). B: MD at six months. C: MD at long-term follow-up period (12 months or more). PV, percutaneous vertebroplasty; BK, balloon kyphoplasty; CI, confidence interval; IV, inverse variance.

**Fig 3:**
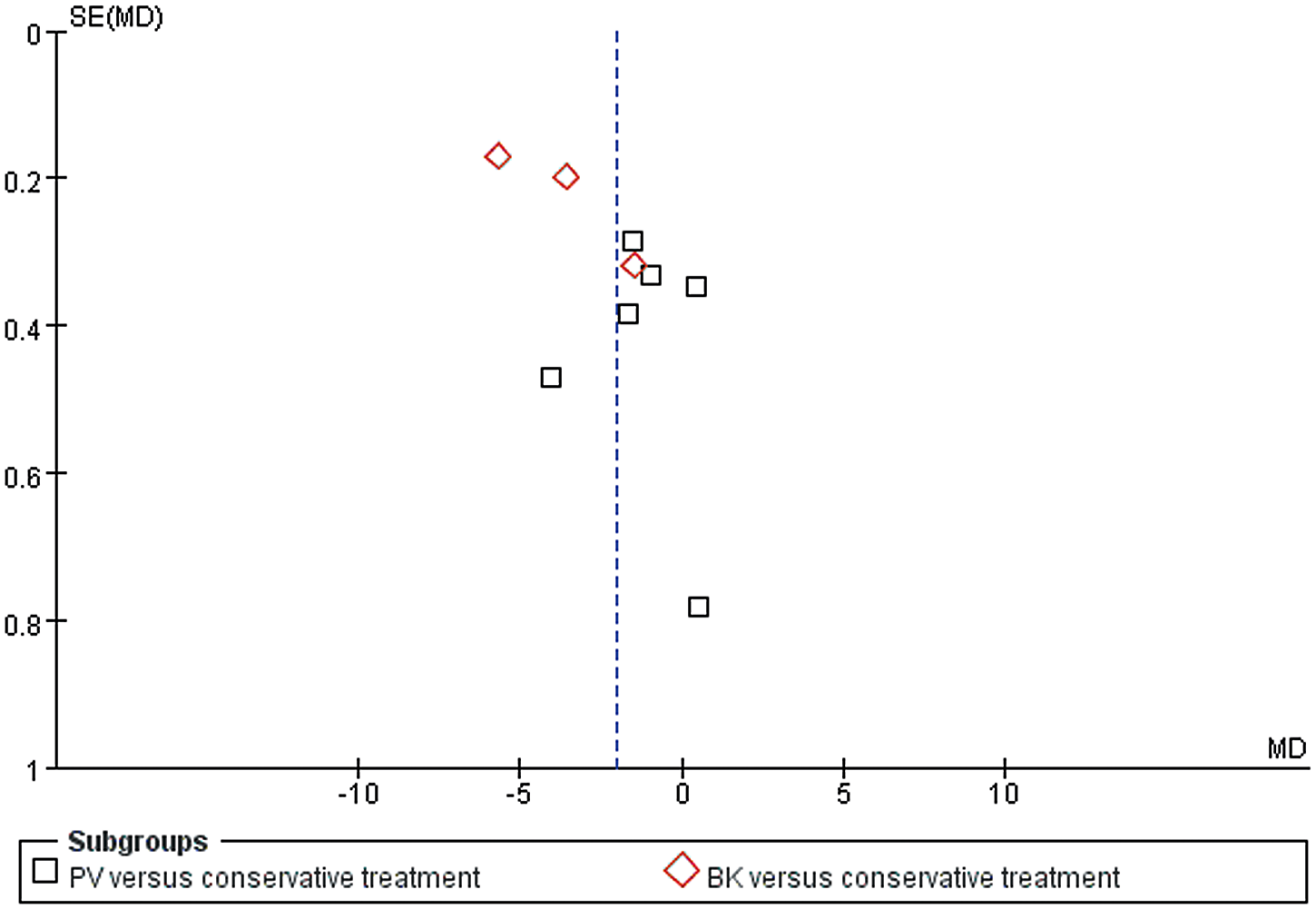
Funnel plot of included studies regarding self-related pain in the short term. Short term: not longer than 3 months. Log of mean difference were plotted against the standard error of mean difference of each study to identify asymmetry in the distribution of trials.

In total, ten trials evaluated self-related pain using a numeric rating scale (NRS) and a visual analog scale (VAS). Among these trials, nine (1,014 patients) reported data at short-term follow-up period, four (597 patients) at mid-term follow-up period and five (604 patients) at long-term follow-up term. We found significant improvements with surgical management for pain relief in the short term (MD −2.05, 95% CI, −3.55 to −0.56; P = 0.007), mid term (MD −1.70, 95% CI, −2.78 to −0.62; P = 0.002) and long term (MD −1.24, 95% CI, −2.20 to −0.29; P = 0.01) (Fig 2). In the test for subgroup differences, PV was seen to be more effective than conservative treatment at short-term (MD −1.28, 95% CI, −2.42 to −0.13; P = 0.03) (Fig 2A), mid-term (MD −1.79, 95% CI, −3.38 to −0.20; P = 0.03) (Fig 2B), and long-term follow-up periods (MD −1.38, 95% CI, −2.64 to −0.12; P = 0.03) (Fig 2C) in reducing pain. We only found significant improvements with BK for pain relief in the short term (MD −3.60, 95% CI, −5.80 to −1.39; P = 0.001) (Fig 2A). In addition, it can be observed that the funnel plot had an asymmetrical distribution regarding self-related pain at short-term follow-up period (Fig 3). There was a suggestion of publication bias according to the shape of the funnel plot.

### Adverse events

**Fig 4:**
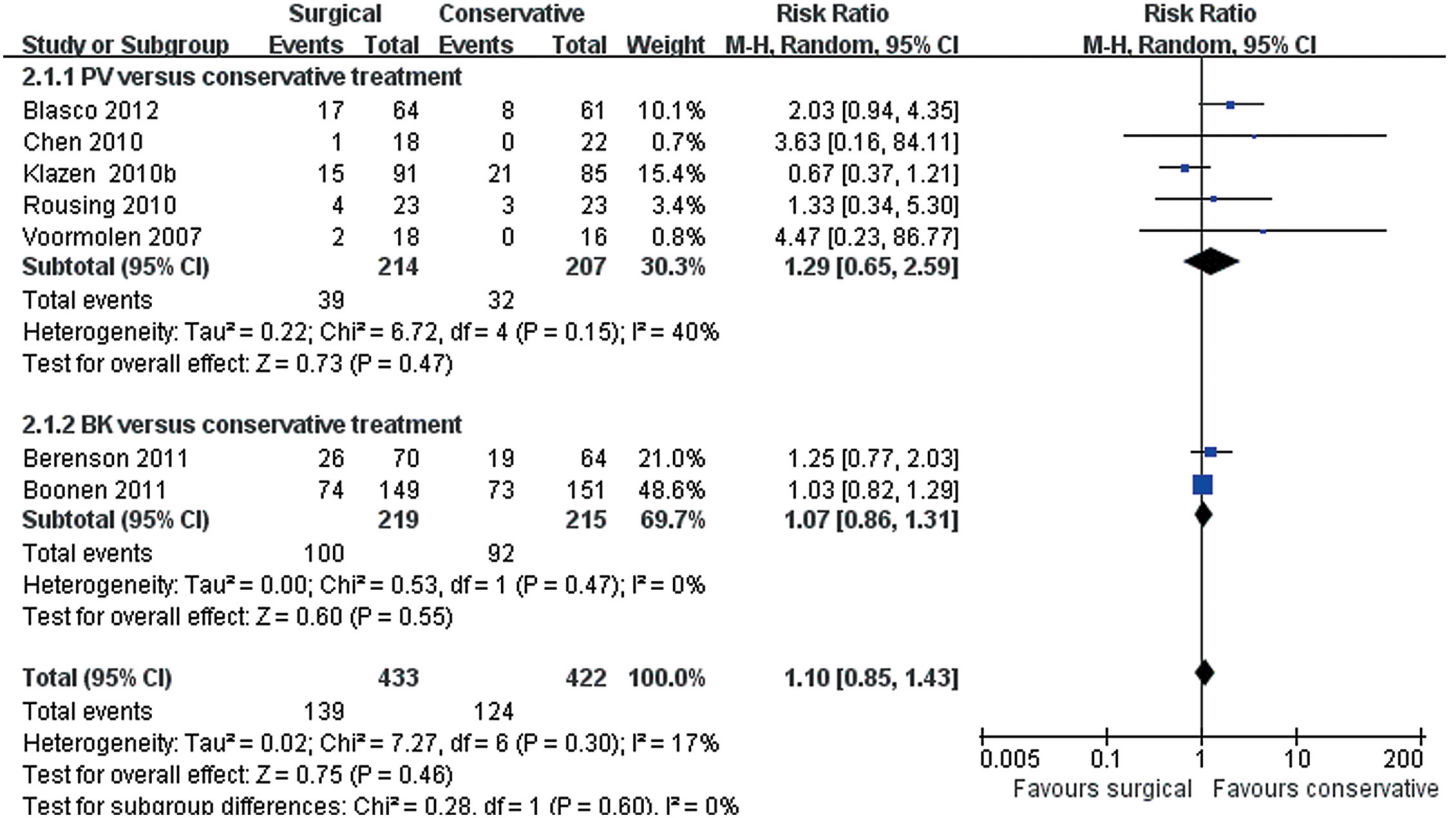
Overall adverse events for surgical versus conservative treatment. Markers represent point estimates of risk ratios, marker size represents study weight in random-effects meta-analysis. Horizontal bars indicate 95% confidence intervals. CI, confidence interval; M-H, mantel-haenszel.

**Fig 5:**
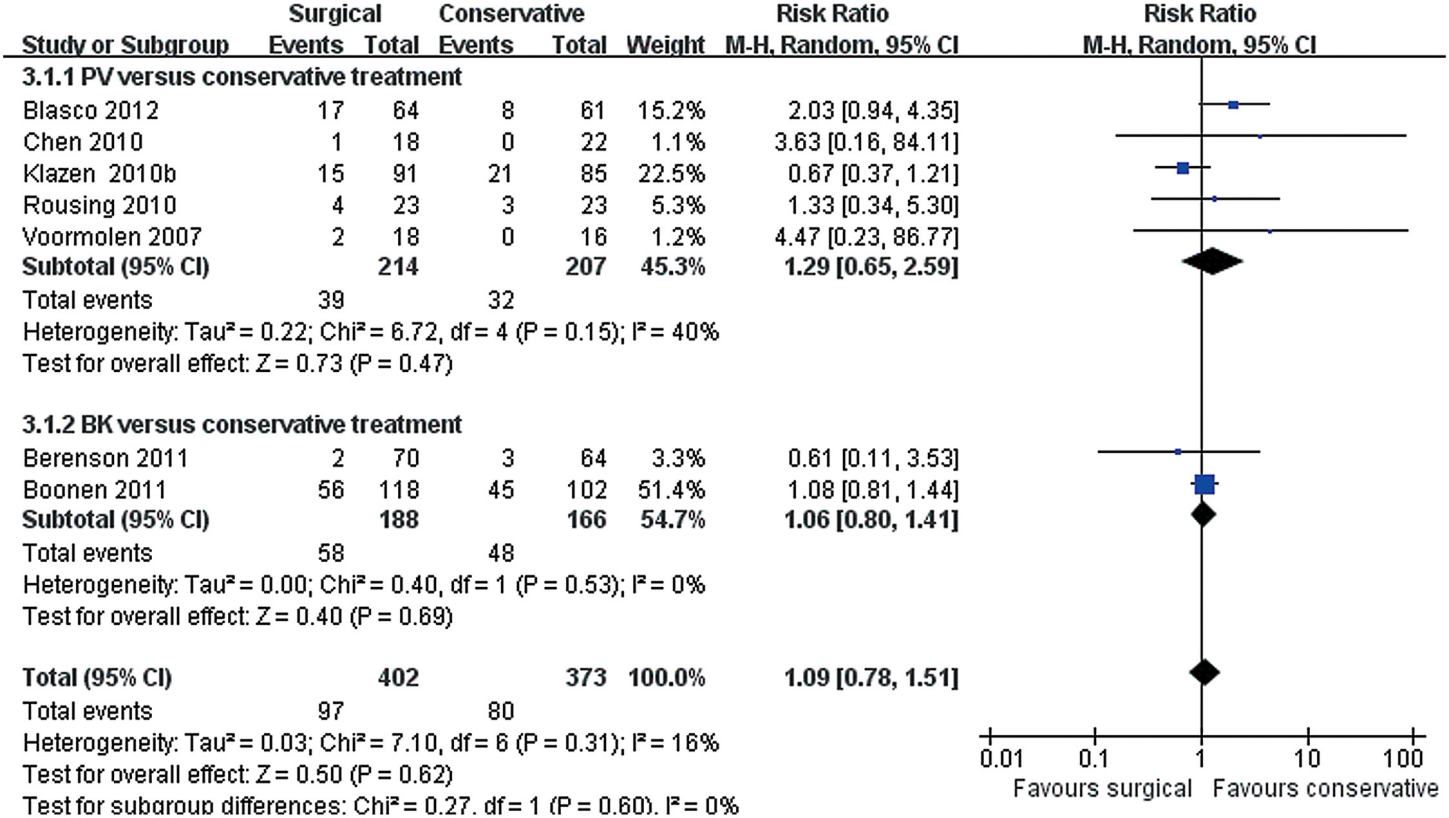
Incidence of new fractures for surgical versus conservative treatment.

Overall, no significant difference between surgical and conservative treatments was observed for adverse events from seven trials consisting of 855 patients (139/433 versus 124/422; RR 1.10, 95% CI, 0.85 to 1.43; P = 0.46) (Fig 4). In the test for subgroup differences, five trials reported no significant difference between PV and conservative treatments(39/214 versus 32/207; RR 1.29, 95% CI, 0.65 to 2.59; P = 0.47), and two reported no significant difference between BK and conservative treatments(100/219 versus 92/215; RR 1.07, 95% CI, 0.86 to 1.31; P = 0.55). One trial was not entered into this forest plot because of incomplete data. The study did not report adverse events in the control group [38]. Further analyses by types of adverse events showed that musculoskeletal disorders were the most common adverse events in both groups. Among musculoskeletal disorders, the frequency of new fractures was the highest (97/402 versus 80/373), but no statistically significant difference was observed between groups (P = 0.62) (Fig 5).

For individual adverse events, no statistically significant difference between the surgical and conservative treatment groups was observed (S3 Fig). One study [32] stated that adverse events related to surgical procedure include hematoma and urinary tract infection. Other adverse events that have been reported include infections, cardiovascular and vascular disorders, injury or procedural complications, and blood and lymphatic disorders.

### Function or disability

**Fig 6:**
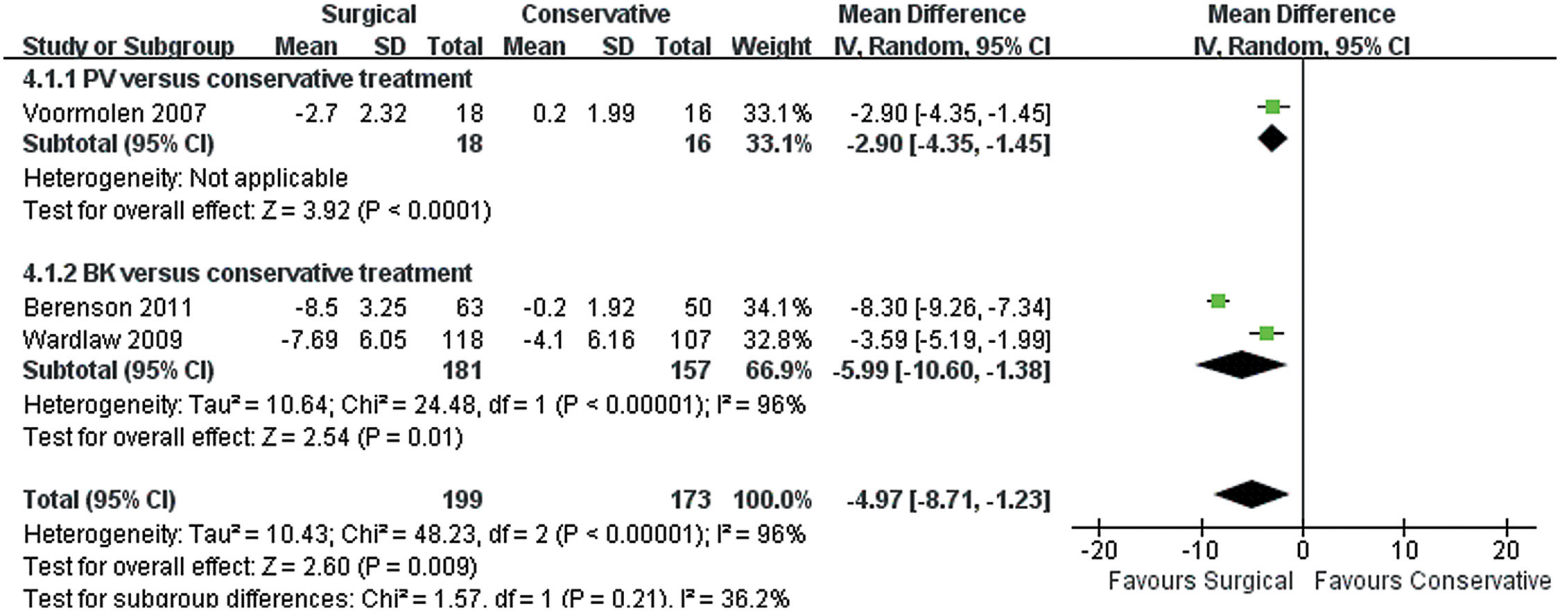
RMD score for surgical versus conservative treatment in the short term. Short term: not longer than 3 months.

Four trials collected data for the Roland—Morris Disability (RMD) score, with three (372 patients) reporting a decrease in the RMD score after surgical intervention at short-term follow-up period (MD −4.97, 95% CI, −8.71 to −1.23; P = 0.009) (Fig 6). In the test for subgroup differences, RMD score decreased with BK compared with that with conservative treatment (MD -5.99, 95% CI, -10.60 to -1.38; P = 0.01). Only one trial [28] at six months follow-up was noted, and surgical treatment was more effective in disability on the RMD score. However, no significant differences were observed between groups at twenty-four months follow-up.

### Health-related quality of life

We used more than one measure of quality of life, and we preferentially included the following measures: the Short-Form 36 (SF-36), the Quality of Life Questionnaire of the European Foundation for Osteoporosis (QUALEFFO), and the European Quality of Life–5 Dimensions (EQ-5D).

**Fig 7:**
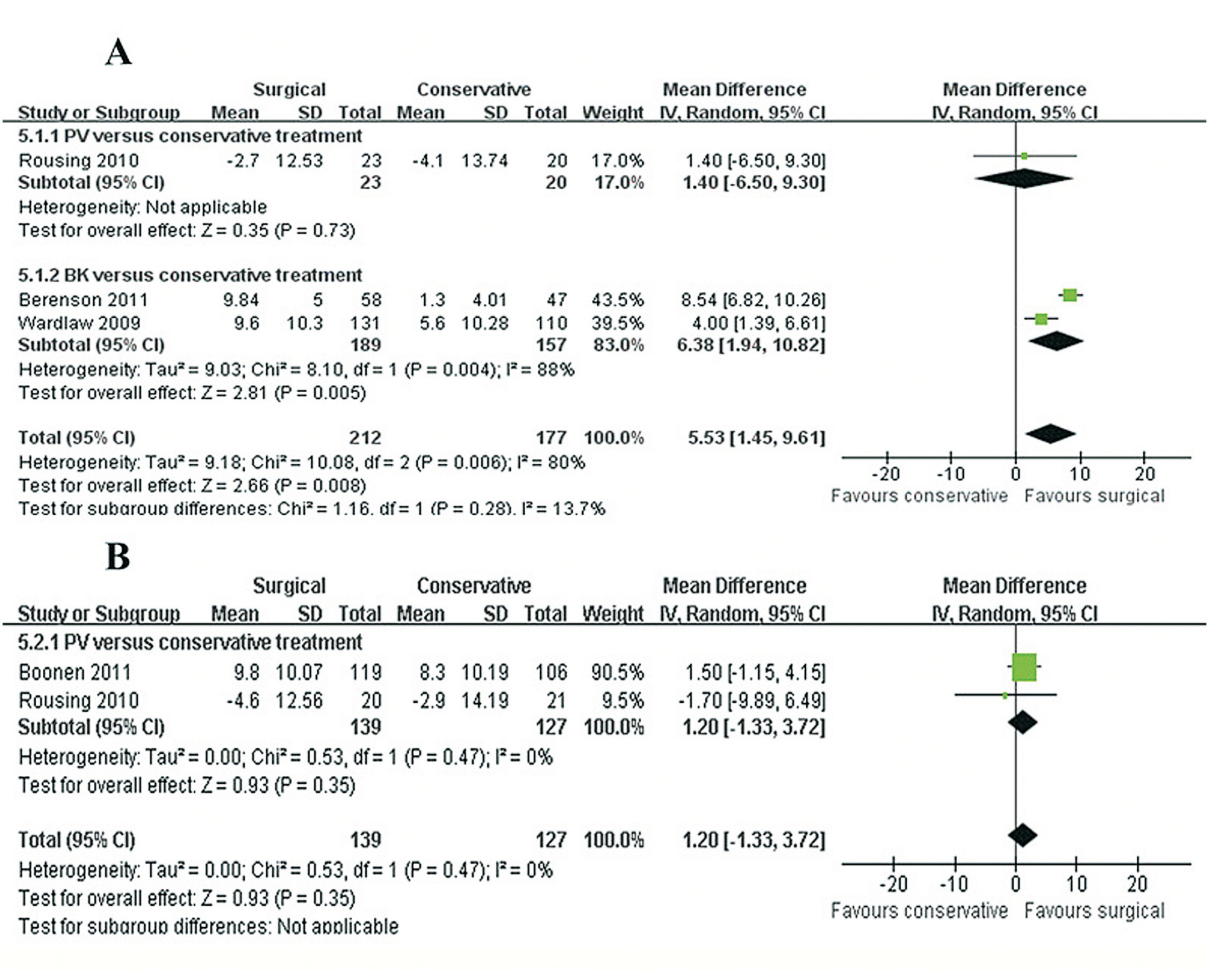
SF-36 PCS score for surgical versus conservative treatment. A: mean difference (MD) at the end of the intervention (not longer than 3 months). B: MD at long-term follow-up period (12 months or more).

SF-36: Four trials, with a total of 614 patients reported data for the Short-Form 36 Physical Component Summary (SF-36 PCS) score. We observed a greater increase at short-term follow-up period after surgical procedure (MD 5.53, 95% CI, 1.45 to 9.61; P = 0.008) (Fig 7A). In the test for subgroup differences, we observed significant differences in favor of BK at short-term follow-up period (MD 7.16, 95% CI, 5.72 to 8.60; P < 0.00001). However, no difference was observed between the two groups at long-term follow-up period (MD 1.20, 95% CI, −1.33 to 3.72; P = 0.93) (Fig 7B). A single trial [25] reported that surgical treatment was more effective in improving quality of life on the SF-36 PCS score at six months follow-up. Considering the Short-Form 36 Mental Component Summary (SF-36 MCS) score, only small study effects were found in favor of the surgical group at short-term follow-up period (MD 7.38, 95% CI, 1.13 to 13.63; P = 0.02) (S4 Fig).

QUALEFFO: Two trials (144 patients) collected data for the QUALEFFO score, which reported that surgical treatment was more effective than conservative treatment at short-term follow-up period (MD −5.01, 95% CI, −8.11 to −1.91; P = 0.002) (S5 Fig). A single trial [39] reported that significant improvement was obtained at both six and twelve months follow-up in the surgical group.

EQ-5D: Three trials, with a total of 500 patients reported data for EQ-5D. However, no significant differences were observed between surgical and conservative management for the EQ-5D score at short- or long-term follow-up periods. (S6 Fig).

### Hospitalization costs

Two trials [29, 32] reported hospitalization costs. Data from Klazen [32] showed that the cost of surgery in the first month was higher than the cost of conservative treatment because of procedural costs (P < 0.0001). However, the difference was no longer significant at one year (P = 0.087), which resulted from continued pain and loss of function in the conservative treatment group. The resulting incremental cost-effectiveness suggested that vertebroplasty was warranted for the patients with vertebral fractures treated at a mean of 5.6 weeks after the start of symptoms. Data from Fritzell [29] demonstrated that BK costs more than standard medical treatment with a significant difference of 7,833€(95% CI, 1671 to 12,511), and from this trial it was not possible to demonstrate that BK was more cost-effective than standard medical treatment in patients treated for an acute or subacute vertebral fracture because of osteoporosis.

## Comparison 2: Surgical Intervention Versus a sham Surgical Procedure for Treating VCF with Osteopenia

### Self-related pain

**Fig 8:**
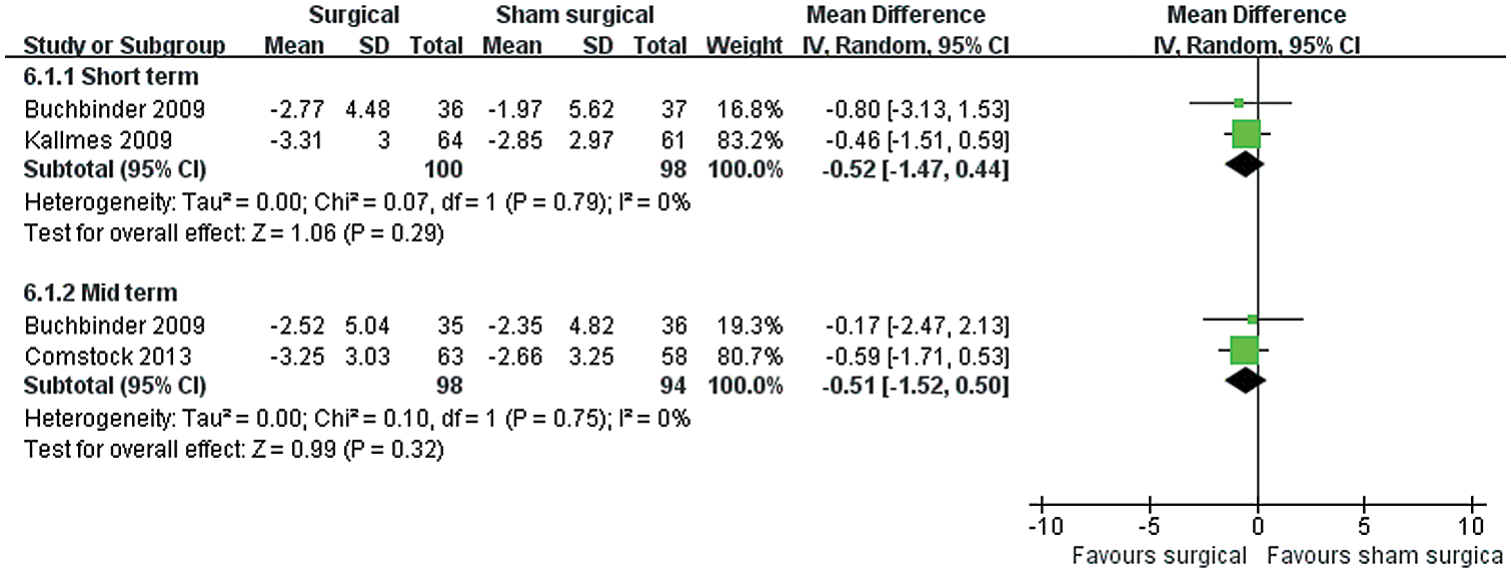
Self-related pain for surgical versus sham surgical treatment. Short term: not longer than 3 months; Mid term: 6 months.

Three trials collected data for self-related pain, with two (198 patients) reporting pain relief after surgical intervention at short-term follow-up period (MD −4.97, 95% CI, −8.71 to −1.23; P = 0.009) (Fig 8). However, no significant differences were found in the mid term. Only one trial [30] at twelve months follow-up period was noted, and there were no significant differences between two groups. Only one surgical method PV was used, so we did not make any subgroup analyses in comparison 2.

### Adverse events

Data on adverse events were available for both studies. No significant difference between surgical intervention and sham surgical intervention was observed in the pooled results for overall adverse events (20/106 versus 16/103; RR 1.32, 95% CI, 0.80 to 2.18; P = 0.28) (S7 Fig). Adverse events in the two studies included incident fracture, injury or procedural complications, tachycardia and rigors, osteomyelitis, tightness in the back or rib cage, and pain [24,25].

### Function or disability

Three trials collected data for the RMD score, and no significant difference was found among groups at short-term (MD 0.03, 95% CI, −1.77 to 1.83; P = 0.98) and mid-term follow-up periods (MD −0.73, 95% CI, −2.76 to 1.30; P = 0.48 (S8 Fig). A single trial [30] reported that there were no significant differences between groups at twelve months follow-up.

### Health-related quality of life

A single trial [25] found no significant difference between the two intervention groups at one month follow-up for the SF-36 PCS score, SF-36 MCS score, and EQ-5D score. Considering the QUALEFFO score, no significant differences were observed between groups at any time points measured, except at one week, which favored the sham procedure group [24].

### Subgroup analysis

The primary comparison in this review was any surgical intervention versus any conservative intervention. Secondary comparison analyses were any surgical intervention versus a sham surgical procedure. The subgroup analysis included two comparisons as follows: PV versus conservative treatment and BK versus conservative treatment. Only one outcome, the self-related pain at short-term follow-up period, showed significant heterogeneity between the treatment effects of the different classes of intervention. In all other cases, threre was no significant heterogeneity between the treatment effects of the different classes of intervention (Table 2). One outlying trial was the cause of this heterogeneity in the self-related pain at short-term follow-up period [37]; when this trial was excluded from the analysis, the result remained significant (MD −1.85, 95% CI, −3.68 to −0.02; P = 0.05), but the test for between subgroup heterogeneity was no longer significant (P = 0.30).

**Table 2:** Summary of results.

Outcomes	No of trials	No of participants	Surgical interventions	Mean difference (95% CI); P-value	Heterogeneity	Subgroup
**Comparison 1: Surgical versus conservative interventions for treating VCF**						
**Pain**						
Short term	9	1014	PV, BK	-2.05 (-3.55 to -0.56), P = 0.007	Chi^2^ = 474.68; I^2^ = 98%; P<0.00001	Chi^2^ = 3.34; I^2^ = 70.1%; P = 0.07
Mid term	4	597	PV, BK	-1.70 (-2.78 to -0.62), P = 0.002	Chi^2^ = 29.88; I^2^ = 90%; P<0.00001	Chi^2^ = 0.12; I^2^ = 0%; P = 0.73
Long term	5	604	PV, BK	-1.24 (-2.20 to -0.29), P = 0.01	Chi^2^ = 28.46; I^2^ = 86%; P<0.0001	Chi^2^ = 0.91; I^2^ = 0%; P = 0.34
**Adverse events**	7	855	PV, BK	1.10 (0.85 to 1.43), P = 0.46	Chi^2^ = 7.27; I^2^ = 17%; P = 0.30	Chi^2^ = 0.28; I^2^ = 0%; P = 0.60
**New fractures**	7	775	PV, BK	1.09 (0.78 to 1.51), P = 0.62	Chi^2^ = 7.10; I^2^ = 16%; P = 0.31	Chi^2^ = 0.27; I^2^ = 0%; P = 0.60
**RMD**						
Short term	3	372	PV, BK	-4.97 (-8.71 to -1.23), P = 0.009	Chi^2^ = 48.23; I^2^ = 96%; P<0.00001	Chi^2^ = 1.57; I^2^ = 36.2%; P = 0.21
**SF-36 PCS**						
Short term	3	389	PV, BK	5.53 (1.45 to 9.61), P = 0.008	Chi^2^ = 10.08; I^2^ = 80%; P = 0.006	Chi^2^ = 1.16; I^2^ = 13.7%; P = 0.28
Long term	2	266	PV, BK	1.20 (-1.33 to 3.72), P = 0.35	Chi^2^ = 0.53; I^2^ = 0%; P = 0.47	
**SF-36 MCS**						
Short term	2	148	PV, BK	7.38 (1.13 to 13.63), P = 0.02	Chi^2^ = 2.06; I^2^ = 51%; P = 0.15	
**EQ-5D**						
Short term	2	274	PV, BK	0.05 (-0.13 to 0.23), P = 0.60	Chi^2^ = 2.11; I^2^ = 53%; P = 0.15	
Long term	2	258	PV, BK	-0.01 (-0.26 to 0.23), P = 0.91	Chi^2^ = 3.83; I^2^ = 74%; P = 0.05	
**QUALEFFO**						
Short term	2	144	PV, BK	-5.01 (-8.11 to -1.91), P = 0.002	Chi^2^ = 1.28; I^2^ = 22%; P = 0.26	
**Comparison 2: Surgical intervention versus a sham surgical procedure for treating VCF**						
**Pain**						
Short term	2	198	PV	-0.52 (-1.47 to 0.44), P = 0.29	Chi^2^ = 0.07; I^2^ = 0%; P = 0.79	
Mid term	2	192	PV	-0.51 (-1.52 to 0.50), P = 0.32	Chi^2^ = 0.10; I^2^ = 0%; P = 0.75	
**RMD**						
Short term	2	198	PV	0.03 (-1.77 to 1.83), P = 0.98	Chi^2^ = 0.28; I^2^ = 0%; P = 0.60	
Mid term	2	192	PV	-0.73 (-2.76 to 1.30), P = 0.48	Chi^2^ = 0.16; I^2^ = 0%; P = 0.69	
**Adverse events**	2	209	PV	1.32 (0.80 to 2.18), P = 0.28	Chi^2^ = 0.07; I^2^ = 0%; P = 0.80	

**Abbreviations** VCF: vertebral compression fracture; CI: confidence interval; Short term: not longer than 3 months; Mid term: 6 months; Long term: 12 months or more; Chi^2^: Chi-squared test, assessment for heterogeneity; I^2^ describes the proportion of variation estimated to be due to heterogeneity.

## Discussion

Previous reviews have examined the results of PV versus conservative treatment or BK versus conservative treatment, but none has combined the surgical methods. BK is a type of PV, and they invovle similar techniques. In addition, both of them have less invasive procedures with similar risk profiles compared to open surgery. Therefore, this review collated all the evidence from a large number of trials, which evaluated vertebroplasty and kyphoplasty into one review to assess the overall effect of surgical versus non-surgical treatment of VCFs with osteopenia. It also allowed an indirect comparison of between PV and BK, and provided no evidence of differences in the treatment effect between the two types of surgical methods.

Statistical significance was reported in comparison one for five outcomes: self-related pain, RMD, SF-36 PCS, SF-36 MCS, and QUALEFFO. The most of the significant differences we observed were at short-term follow-up period. Only the improvements of self-related pain were seen in the short, mid, and long terms. This meta-analysis suggested a favorable association of surgical treatment in reducing pain, improving function and quality of life compared with conservative treatment. By contrast, when we focused on the two studies [24, 25] in which surgical procedure was compared with sham procedure, we saw no evidence of improvement in pain relief, physical function and quality of life. The kind of these two studies is generally speaking difficult to achieve in surgical procedures, and often reaches the limit of ethically acceptable studies. Both studies are multicenter, double-blind RCTs published in the New England Journal of Medicine. The results from the two papers have aroused worldwide attention and intense controversy [40–42]. The placebo effect may be explained by several reasons, such as narcotic effects, psychological effects, and neural excitatory effects. However, at long-term follow-up period, pain relief with placebo treatment was similar to that with surgical management. Samuel Butler [40] stated that these two studies comprised samples fewer than 300 patients in both the treatment and control groups, and many patients were unwilling to accept randomization, especially for patients in severe pain. Thus, the small size of sample and selection bias made these studies untrustworthy. In spite of these limitations, we still observed a statistical trend, which suggested that vertebroplasty was more effective than sham treatment. For example, data from Kallmes [25] showed a higher rate of clinically meaningful improvement in pain relief in the vertebroplasty group than that in the control group (64% versus 48%, P = 0.06). Vertebroplasty may be effective with a larger sample size. Therefore, high-quality, well-designed studies are needed to address the areas of uncertainty.

The incidence of new fractures was similar after surgical and conservative therapy. Thus, the occurrence of new vertebral fractures was due to ongoing osteoporosis and not the type of therapy. About 20% of patients with a history of osteoporotic vertebral fractures will experience a new vertebral fracture within a year, depending on the severity of the prior fracture [43]. In addition, no statistically significant difference was observed between groups in other adverse events. In this meta-analysis, we did not believe that the risk of adverse events after surgery should be a contraindication for the operation.

The findings from our review were consistent with findings from previous reviews. Five reviews have been published, and all of them included trials only using vertebroplasty as the experimental group [16–19, 44]. Four of these reviews [16–17, 19, 44] restricted the results to RCTs only, whereas one [18] included randomized and non-randomized clinical trials. Three of these reviews [16, 17, 19] found that vertebroplasty in pain relief is more obvious than that in conservative treatment at short-term, mid-term, and long-term follow-up. Four trials [16, 17, 19, 44] compared vertebroplasty with a sham procedure in patients with VCFs, and all failed to show an advantage of vertebroplasty over placebo.

### Strengths

Our study had several strengths. The review highlights the wide range of surgical techniques being used in the treatment of VCFs with osteopenia, and previous reviews focused on one type of surgical management. We made subgroup analyses for vertebroplasty and kyphoplasty, and it allowed an indirect comparison between them. Our review methods were systematic and exhaustive. We employed a broad search strategy without any publication dates or language restrictions. Relevant citations in selected articles were also examined. All trials included in our review were RCTs. Study selection, quality assessment, and data extraction were performed by at least two independent reviewers. We mapped all possible treatment comparisons, and six outcomes were reported in this meta-analysis. Some heterogeneity was observed in the meta-analysis, so we generated random-effects models that accounted for between-study variability.

### Limitations

First, all studies included in this review used pain scales. However, different studies used different pain surveys as outcomes, which possibly created some of the heterogeneity we observed among trials. Second, we noted minimal focus on hospitalization cost analysis of the surgical intervention; therefore, little is known about the cost-effectiveness and economic value of these surgical managements. Third, we estimated the standard deviations for several studies using the formula suggested in the Cochrane Handbook for Systematic Reviews of Interventions because a response was unavailable from certain authors.

### Implications for Research

Evidence of better quality and from a larger sample size is required before a recommendation can be made. Further RCTs are needed until we can establish whether surgical management is more effective than non-surgical management in patients with VCFs due to osteopenia. Future studies should identify which type of surgical treatment provides more advantages. The shortcomings of the present evidence in this review include small study size, selection bias, performance bias, detection bias, and attrition bias. Future trials should be reported following the CONSORT guidelines [45], and data should be analyzed according to intention to treat principles. This review illustrated the need for the universal use of relevant outcome measures, especially adverse events and hospitalization cost outcomes. Moreover, systematic data collection at short, mid-, and long-term follow-up is essential.

## Supporting Information

S1 Checklist PRISMA Checklist of this meta-analysis. (DOC)

S1 File Search strategies for all databases. (DOC)

S1 Fig Risk of bias graph using the Cochrane Risk of Bias tool. (TIF)

S2 Fig Risk of bias summary using the Cochrane Risk of Bias tool. (TIF)

S3 Fig Individual adverse events for surgical versus conservative treatment. Markers represent point estimates of risk ratios, marker size represents study weight in random-effects meta-analysis. Horizontal bars indicate 95% confidence intervals. CI, confidence interval; M-H, mantel-haenszel.
(TIF)

S4 Fig SF-36 MCS score for surgical versus conservative treatment in the short term. Short term: not longer than 3 months; Markers represent point estimates of mean difference, marker size represents study weight in random-effects meta-analysis. Horizontal bars indicate 95% confidence intervals. CI, confidence interval; IV, inverse variance.
(TIF)

S5 Fig QUALEFFO score for surgical versus conservative treatment in the short term. Short term: not longer than 3 months; Markers represent point estimates of mean difference, marker size represents study weight in random-effects meta-analysis. Horizontal bars indicate 95% confidence intervals. CI, confidence interval; IV, inverse variance.
(TIF)

S6 Fig EQ-5D score for surgical versus conservative treatment in the short and long terms. Short term: not longer than 3 months; Long term: 12 months or more; Markers represent point estimates of mean difference, marker size represents study weight in random-effects meta-analysis. Horizontal bars indicate 95% confidence intervals. CI, confidence interval; IV, inverse variance.
(TIF)

S7 Fig Overall adverse events for surgical versus sham surgical treatment. Markers represent point estimates of risk ratios, marker size represents study weight in random-effects meta-analysis. Horizontal bars indicate 95% confidence intervals. CI, confidence interval; M-H, mantel-haenszel.
(TIF)

S8 Fig RMD score for surgical versus sham surgical treatment in the short and mid terms. Short term: not longer than 3 months; Mid term: 6 months; Markers represent point estimates of mean difference, marker size represents study weight in random-effects meta-analysis. Horizontal bars indicate 95% confidence intervals. CI, confidence interval; IV, inverse variance.
(TIF)
