# Supplementary material for: Surgical Versus Non-Surgical Treatment for Vertebral Compression Fracture with Osteopenia: A Systematic Review and Meta-Analysis
Source: PLoS One. 2015 May 28;10(5):e0127145. doi: 10.1371/journal.pone.0127145 (PMC4447413; doi:10.1371/journal.pone.0127145)
Supplement: S1 File — (DOC) [file pone.0127145.s002.doc]

**Appendix1. Database search strategies**

**1.Cochrane Central Register of Controlled Trials (CENTRAL) in The Cochrane Library**

#1 MeSH descriptor: [Spine] explode all trees

#2 vertebra*:ti,ab,kw or spin*:ti,ab,kw or spinal column:ti,ab,kw or vertebral column:ti,ab,kw or backbone:ti,ab,kw or thoracic vertebrae:ti,ab,kw or lumbar vertebrae:ti,ab,kw or thoracolumbar vertebrae:ti,ab,kw in Trials (Word variations have been searched)

#3 #1 OR #2 **(16883)**

#4 MeSH descriptor: [Fractures, Bone] explode all tree

#5 fracture*:ti,ab,kw in Trials (Word variations have been searched)

#6 #4 OR #5 **(9400)**

#7 MeSH descriptor: [Vertebroplasty] explode all trees

#8 surg*: ab,ti,kw OR operat*: ab,ti,kw OR invasive: ab,ti,kw OR vertebroplasty: ab,ti,kw OR kyphoplasty: ab,ti,kw OR pedicle screw fixation: ab,ti,kw OR pedicle screws system: ab,ti,kw OR anterior reconstruction: ab,ti,kw OR anterior fusion: ab,ti,kw OR posterior reconstruction: ab,ti,kw OR posterior fusion: ab,ti,kw in Trials (Word variations have been searched)

#9 non-surg*: ab,ti,kw OR nonsurg*: ab,ti,kw OR non-operat*: ab,ti,kw OR nonoperat*: ab,ti,kw OR non-invasive: ab,ti,kw OR noninvasive: ab,ti,kw OR conservative treatment: ab,ti,kw OR conservative therapy: ab,ti,kw OR conservative intervention: ab,ti,kw OR bed rest: ab,ti,kw OR physical therapy: ab,ti,kw OR physiotherapy : ab,ti,kw OR medication: ab,ti,kw OR analgesics: ab,ti,kw OR brace: ab,ti,kw OR bracing: ab,ti,kw OR cast: ab,ti,kw OR rehabilitation: ab,ti,kw in Trials (Word variations have been searched)

#10 #7 OR #8 OR #9 **(156662)**

#11 #3 AND #6 AND #10 **(644)**

**2. PubMed**

#1 "Single-Blind Method"[Mesh] OR "Double-Blind Method"[Mesh] OR "Randomized Controlled Trials as Topic"[Mesh] OR "Randomized Controlled Trial" [Publication Type] OR "Intention to Treat Analysis"[Mesh] OR "Controlled Clinical Trials as Topic"[Mesh] OR "Clinical Trials as Topic"[Mesh] OR "Clinical Trial" [Publication Type]

#2 "random*"[Text Word] OR allocation[Text Word] OR "random allocation"[Text Word] OR placebo[Text Word] OR single blind[Text Word] OR double blind[Text Word] OR "randomized controlled trial*"[Text Word] OR RCT[Text Word]

#3 randomized controlled trial[Publication Type]

#4 #1 OR #2 OR #3 **(1426009)**

#5 Spine[Mesh]

#6 vertebra*[Title/Abstract] OR spin*[Title/Abstract] OR spinal column[Title/Abstract] OR vertebral column[Title/Abstract] OR backbone[Title/Abstract] OR thoracic vertebrae[Title/Abstract] OR lumbar vertebrae[Title/Abstract] OR thoracolumbar vertebrae[Title/Abstract]

#7 #5 OR #6 **(555923)**

#8 Fractures, Bone[Mesh]

#9 fracture*[Title/Abstract]

#10 #8 OR #9 **(217221)**

#11 Vertebroplasty [Mesh]

#12 surg*[Title/Abstract] OR operat*[Title/Abstract] OR invasive[Title/Abstract] OR vertebroplasty[Title/Abstract] OR kyphoplasty[Title/Abstract] OR pedicle screw fixation[Title/Abstract] OR pedicle screws system[Title/Abstract] OR anterior reconstruction[Title/Abstract] OR anterior fusion[Title/Abstract] OR posterior reconstruction[Title/Abstract] OR posterior fusion[Title/Abstract]

#13 non-surg*[Title/Abstract] OR nonsurg*[Title/Abstract] OR non-operat*[Title/Abstract] OR nonoperat*[Title/Abstract] OR non-invasive[Title/Abstract] OR noninvasive[Title/Abstract] OR conservative [treatment](javascript:void(0);) [Title/Abstract] OR conservative therapy[Title/Abstract] OR conservative intervention[Title/Abstract] OR bed rest[Title/Abstract] OR physical therapy[Title/Abstract] OR physiotherapy[Title/Abstract] OR medication [Title/Abstract] OR analgesics[Title/Abstract] OR brace[Title/Abstract] OR bracing[Title/Abstract] OR cast[Title/Abstract] OR rehabilitation[Title/Abstract]

#14 #11 OR #12 OR #13 **(2298184)**

#15 humans[Species]

#16 #4 AND #7 AND #10 AND #14 AND #15 **(1185)**

**3. Web of Science**

#1 TS=("random*" OR allocation OR "random allocation" OR placebo OR single blind OR double blind OR "randomized controlled trial*" OR "randomised controlled trial*" OR "RCT" OR "clinical trial*") **(1033668)**

#2 TS=(vertebra* OR spin* OR spinal column OR vertebral column OR backbone OR [thoracic](javascript:void(0);) [vertebra](javascript:void(0);)e OR [lumbar](javascript:void(0);) [vertebra](javascript:void(0);)e OR thoracolumbar vertebrae) **(574123)**

#3 TS=(fractures, bone OR fracture*) **(179838)**

#4 TS=( surg* OR operat* OR invasive OR vertebroplasty OR kyphoplasty OR pedicle screw fixation OR pedicle screws system OR anterior reconstruction OR anterior fusion OR posterior reconstruction OR posterior fusion OR non-surg* OR nonsurg* OR non-operat* OR nonoperat* OR non-invasive OR noninvasive OR conservative [treatment](javascript:void(0);) OR conservative therapy OR conservative intervention OR bed rest OR physical therapy OR physiotherapy OR medication OR analgesics OR brace OR bracing OR cast OR rehabilitation) **(1838103)**

#5 #1 AND #2 AND #3 AND #4 **(1601)**

Time span=All years. Databases=SCI-EXPANDED, SSCI, A&HCI, CPCI-S, CPCI-SSH.

**4. EMBASE**

#1 'randomization'/exp OR 'placebo'/exp OR 'placebo effect'/exp OR 'single blind procedure'/exp OR 'double blind procedure'/exp OR 'randomized controlled trial'/exp OR 'randomized controlled trial (topic)'/exp OR 'controlled clinical trial'/exp OR 'controlled clinical trial (topic)'/exp OR 'clinical trial'/exp OR 'clinical trial (topic)'/exp

#2 random*:ab,ti OR allocation:ab,ti OR 'random allocation':ab,ti OR placebo:ab,ti OR 'single blind':ab,ti OR "double blind":ab,ti OR 'randomised controlled trial':ab,ti OR 'randomized controlled trial':ab,ti OR RCT:ab,ti

#3 #1 OR #2 **(1503876)**

#4 'Spine'/exp

#5 vertebra*:ab,ti OR spin*:ab,ti OR 'spinal column':ab,ti OR 'vertebral column':ab,ti OR backbone:ab,ti OR 'thoracic vertebrae':ab,ti OR 'lumbar vertebrae':ab,ti OR 'thoracolumbar vertebrae':ab,ti

#6 #4 OR #5 **(531461)**

#7  'fractures, bone'/exp

#8 fracture*:ab,ti

#9 #7 OR # 8 **(189110)**

#10 'vertebroplasty'/exp

#11 surg*:ab,ti OR operat*:ab,ti OR invasive:ab,ti OR vertebroplasty:ab,ti OR kyphoplasty:ab,ti OR 'pedicle screw fixation':ab,ti OR 'pedicle screws system':ab,ti OR 'anterior reconstruction':ab,ti OR 'anterior fusion':ab,ti OR 'posterior reconstruction':ab,ti OR 'posterior fusion':ab,ti

#12 non-surgery:ab,ti OR 'non surgical':ab,ti OR nonsurg*:ab,ti OR 'non operative':ab,ti OR noninvasive:ab,ti OR 'non invasive':ab,ti OR 'non operation' OR nonoperat*:ab,ti OR 'conservative [treatment](javascript:void(0);)':ab,ti OR 'conservative therapy':ab,ti OR 'conservative intervention':ab,ti OR 'bed rest':ab,ti OR 'physical therapy':ab,ti OR physiotherapy:ab,ti OR medication:ab,ti OR analgesics:ab,ti OR brace:ab,ti OR bracing:ab,ti OR cast:ab,ti OR rehabilitation:ab,ti

#13 #10 OR #11 OR #12 **(2280156)**

#14 #3 AND #6 AND #9 AND #13 **(2019)**

#15 study type：human

#16 #14 AND #15 **(1802)**

**5. Chinese Biomedical Literature Database**

#1  "随机分配"[扩展：不加权] OR "对照组"[扩展：不加权] OR "单盲法"[扩展：不加权] OR "双盲法"[扩展：不加权] OR "随机对照试验"[扩展：不加权] OR "临床对照试验"[扩展：不加权] OR "临床试验"[扩展：不加权]

#2  "随机对照试验(主题)"[扩展：不加权] OR "临床对照试验(主题)"[扩展：不加权] OR "临床试验(主题)"[扩展：不加权]

#3  "随机"[全字段：智能] OR "随机分配"[全字段：智能] OR "随机对照"[全字段：智能] OR "对照"[全字段：智能] OR "盲法"[全字段：智能] OR "单盲"[全字段：智能] OR "双盲"[全字段：智能] OR "随机对照试验"[全字段：智能] OR "随机对照试验（文献类型）"[全字段：智能] OR "随机对照研究"[全字段：智能] OR "临床试验"[全字段：智能] OR "临床观察"[全字段：智能] OR "临床研究"[全字段：智能]

#4  (#1) OR (#2) OR (#3) **(1568909)**

#5  "脊柱"[扩展：不加权]

#6  "脊柱"[中文标题：智能] OR "椎体"[中文标题：智能] OR "胸腰椎"[中文标题：智能] OR "胸椎"[中文标题：智能] OR "腰椎"[中文标题：智能]

#7  "脊柱"[摘要：智能] OR "椎体"[摘要：智能] OR "胸腰椎"[摘要：智能] OR "胸椎"[摘要：智能] OR "腰椎"[摘要：智能]

#8  "脊柱"[关键词：智能] OR "椎体"[关键词：智能] OR "胸腰椎"[关键词：智能] OR "胸椎"[关键词：智能] OR "腰椎"[关键词：智能]

#9  (#5) OR (#6) OR (#7) OR (#8) **(141701)**

#10   “骨折"[扩展：不加权]

#11   “骨折"[中文标题：智能]

#12   "骨折"[摘要：智能]

#13   "骨折"[关键词：智能]

#14   (#10) OR (#11) OR (#12) OR (#13) **(164150)**

#15  "椎体成形术"[扩展：不加权]

#16  "手术"[中文标题：智能] OR "椎体成形术"[中文标题：智能] OR "椎体后凸成形术"[中文标题：智能] OR "椎弓根内固定"[中文标题：智能] OR "椎弓根螺钉内固定"[中文标题：智能] OR "椎弓根螺钉系统"[中文标题：智能] OR "前路融合"[中文标题：智能] OR "后路融合"[中文标题：智能] OR "前路重建"[中文标题：智能] OR "后路重建"[中文标题：智能] OR "非手术"[中文标题：智能] OR "保守治疗"[中文标题：智能] OR "卧床休息"[中文标题：智能] OR "物理治疗"[中文标题：智能] OR "药物治疗"[中文标题：智能] OR "支具"[中文标题：智能] OR "镇痛"[中文标题：智能] OR "腰背肌功能训练"[中文标题：智能]

#17  "手术"[摘要：智能] OR "椎体成形术"[摘要：智能] OR "椎体后凸成形术"[摘要：智能] OR "椎弓根内固定"[摘要：智能] OR "椎弓根螺钉内固定"[摘要：智能] OR "椎弓根螺钉系统"[摘要：智能] OR "前路融合"[摘要：智能] OR "后路融合"[摘要：智能] OR "前路重建"[摘要：智能] OR "后路重建"[摘要：智能] OR "非手术"[摘要：智能] OR "保守治疗"[摘要：智能] OR "卧床休息"[摘要：智能] OR "物理治疗"[摘要：智能] OR "药物治疗"[摘要：智能] OR "支具"[摘要：智能] OR "镇痛"[摘要：智能] OR "腰背肌功能训练"[摘要：智能]

#18  "手术"[关键词：智能] OR "椎体成形术"[关键词：智能] OR "椎体后凸成形术"[关键词：智能] OR "椎弓根内固定"[关键词：智能] OR "椎弓根螺钉内固定"[关键词：智能] OR "椎弓根螺钉系统"[关键词：智能] OR "前路融合"[关键词：智能] OR "后路融合"[关键词：智能] OR "前路重建"[关键词：智能] OR "后路重建"[关键词：智能] OR "非手术"[关键词：智能] OR "保守治疗"[关键词：智能] OR "卧床休息"[关键词：智能] OR "物理治疗"[关键词：智能] OR "药物治疗"[关键词：智能] OR "支具"[关键词：智能] OR "镇痛"[关键词：智能] OR "腰背肌功能训练"[关键词：智能]

#19  (#15) OR (#16) OR (#17) OR (#18) **(987867)**

#20  (#4) AND (#9) AND (#14) AND (#19) **(1915)**

**6. Wanfang Database**

#1  主题:("随机" or "随机分配" or "随机对照" or "对照" or "盲法" or "单盲" or "双盲" or "随机对照试验" or "临床试验" or "临床观察")

#2  主题:("脊柱" or "椎体" or "胸腰椎" or "腰椎" or "胸椎")

#3  主题:("骨折")

#4  主题:("手术" or "椎体成形术" or "椎体后凸成形术" or "椎弓根内固定" or "椎弓根螺钉内固定" or "椎弓根螺钉系统" or "前路融合" or "后路融合" or "前路重建" or "后路重建" or "非手术" or "保守治疗" or "卧床休息" or "物理治疗" or "药物治疗" or "支具" or "镇痛" or "腰背肌功能训练")

#5  (#1) and (#2) and (#3) and(#4) **(1415)**

**7. China National Knowledge Infrastructure**

#1 (SU='随机' OR SU='随机分配' OR SU='随机对照' OR SU='对照' OR SU='盲法' OR SU='单盲' OR SU='双盲' OR SU='随机对照试验' OR SU='随机对照研究' OR SU='临床试验' OR SU='临床观察' OR SU=‘临床研究’)

#2 (SU='手术' OR SU='椎体形成术' OR SU='椎体后凸成形术' OR SU='椎弓根内固定' OR SU='椎弓螺钉内固定' OR SU='椎弓螺钉系统' OR SU='前路融合' OR SU='后路融合' OR SU='前路重建' OR SU='后路重建' OR SU='非手术' OR SU='保守治疗' OR SU='卧床休息' OR SU='物理治疗' OR SU='药物治疗' OR SU='支具' OR SU='镇痛' OR SU='腰背肌功能训练')

#3 (SU='脊柱' OR SU='椎体' OR SU='胸腰椎' OR SU='腰椎' OR SU='胸椎')

#4 (SU='骨折')

#5  #1 并且 #2 并且 #3 并且 #4 **(1812)**
