# Supplementary figures and images for: Surgical Versus Non-Surgical Treatment for Vertebral Compression Fracture with Osteopenia: A Systematic Review and Meta-Analysis
Source: PLoS One. 2015 May 28;10(5):e0127145. doi: 10.1371/journal.pone.0127145 (PMC4447413; doi:10.1371/journal.pone.0127145)

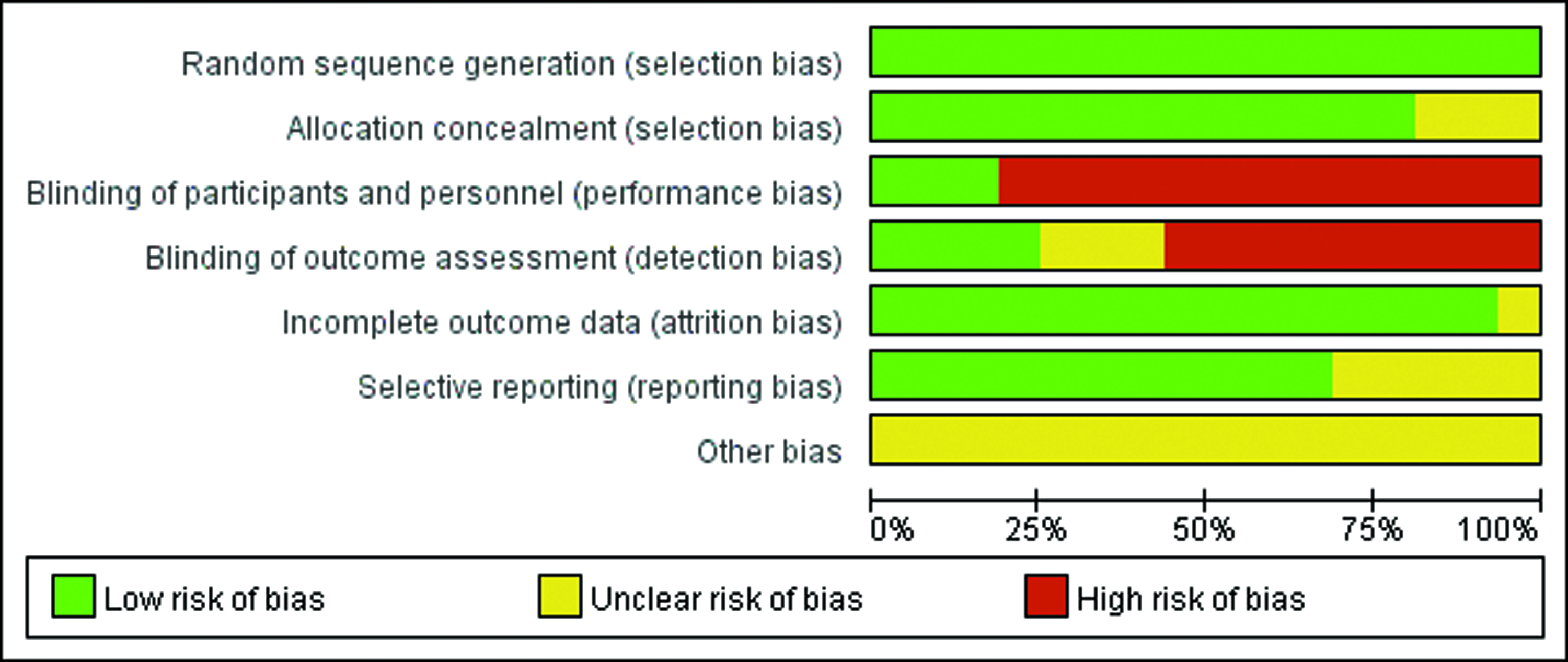

Supplement: S1 Fig — (TIF) [file pone.0127145.s003.tif]

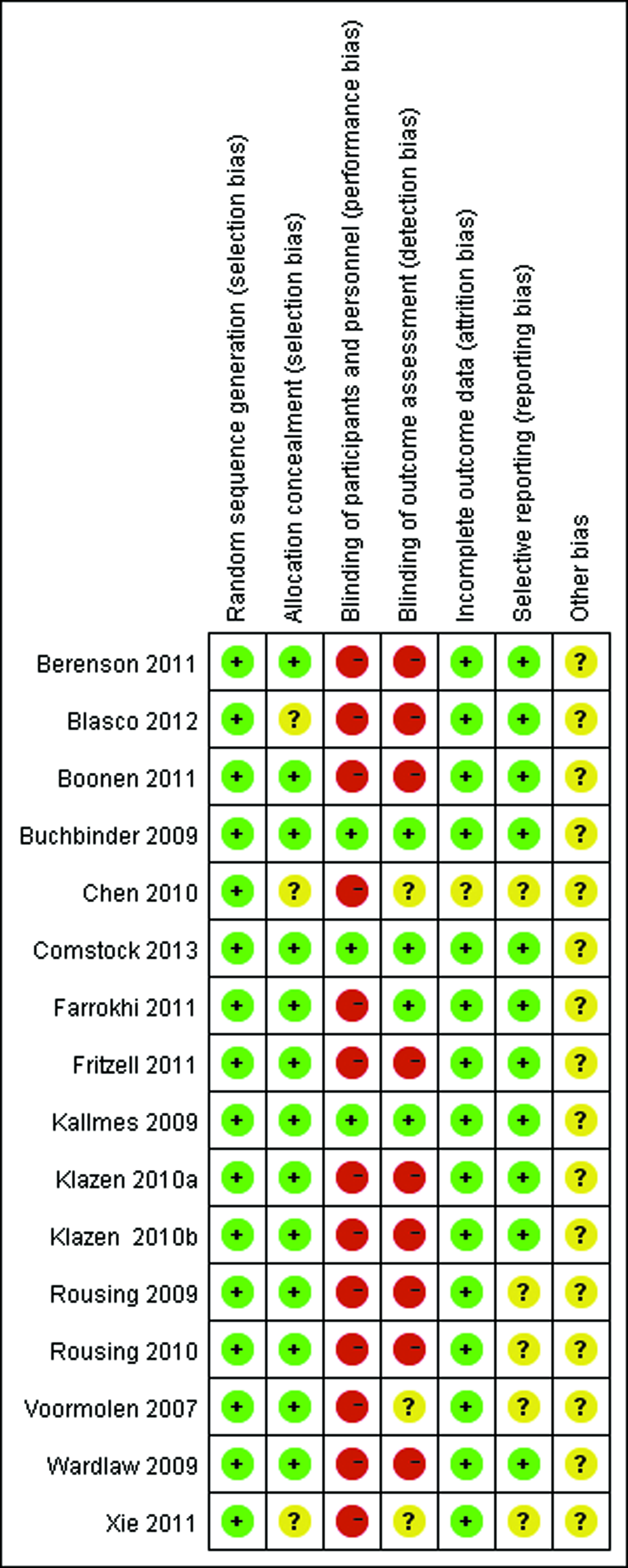

Supplement: S2 Fig — (TIF) [file pone.0127145.s004.tif]

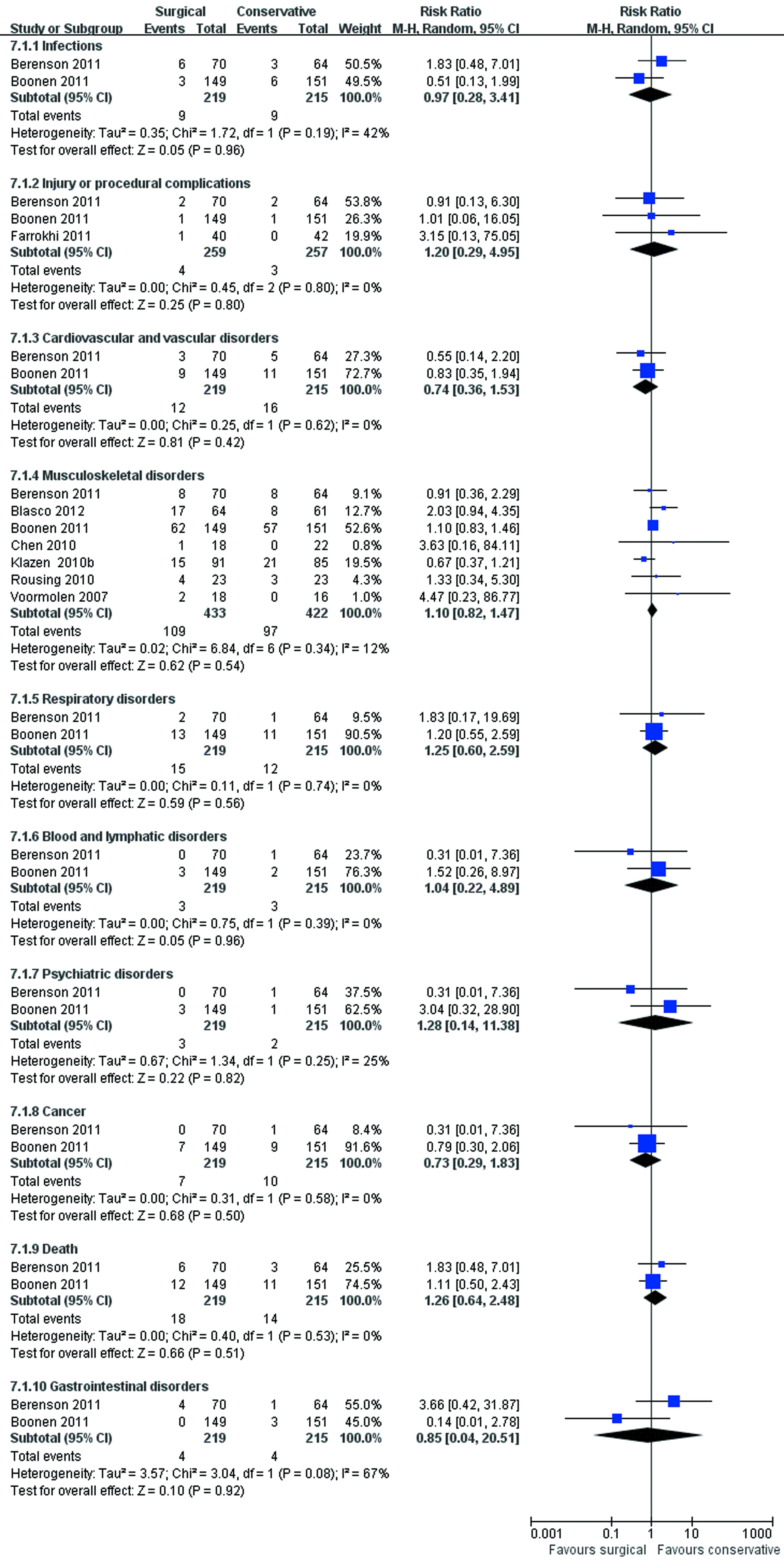

Supplement: S3 Fig — Markers represent point estimates of risk ratios, marker size represents study weight in random-effects meta-analysis. Horizontal bars indicate 95% confidence intervals. CI, confidence interval; M-H, mantel-haenszel. (TIF) [file pone.0127145.s005.tif]

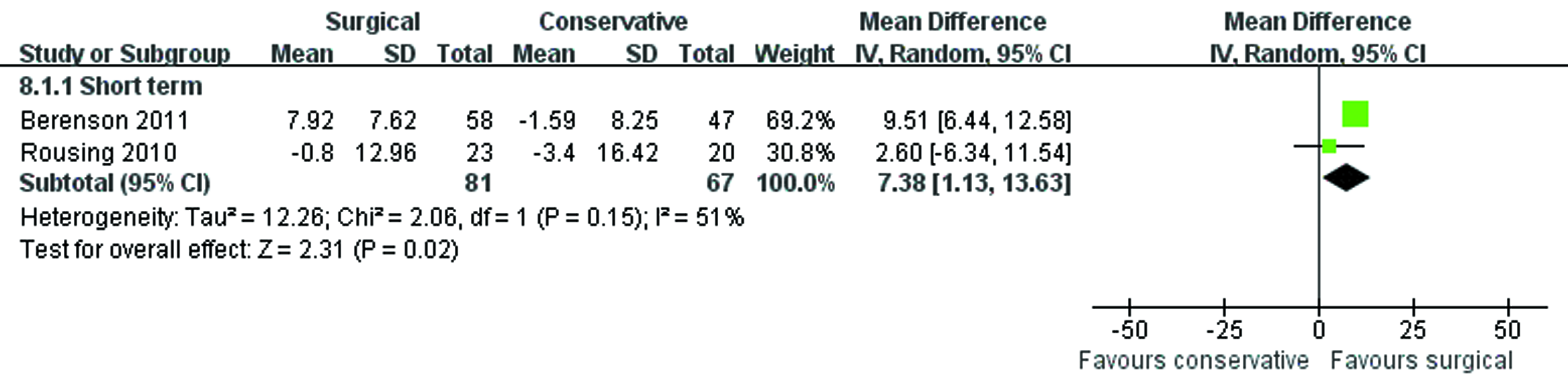

Supplement: S4 Fig — Short term: not longer than 3 months; Markers represent point estimates of mean difference, marker size represents study weight in random-effects meta-analysis. Horizontal bars indicate 95% confidence intervals. CI, confidence interval; IV, inverse variance. (TIF) [file pone.0127145.s006.tif]

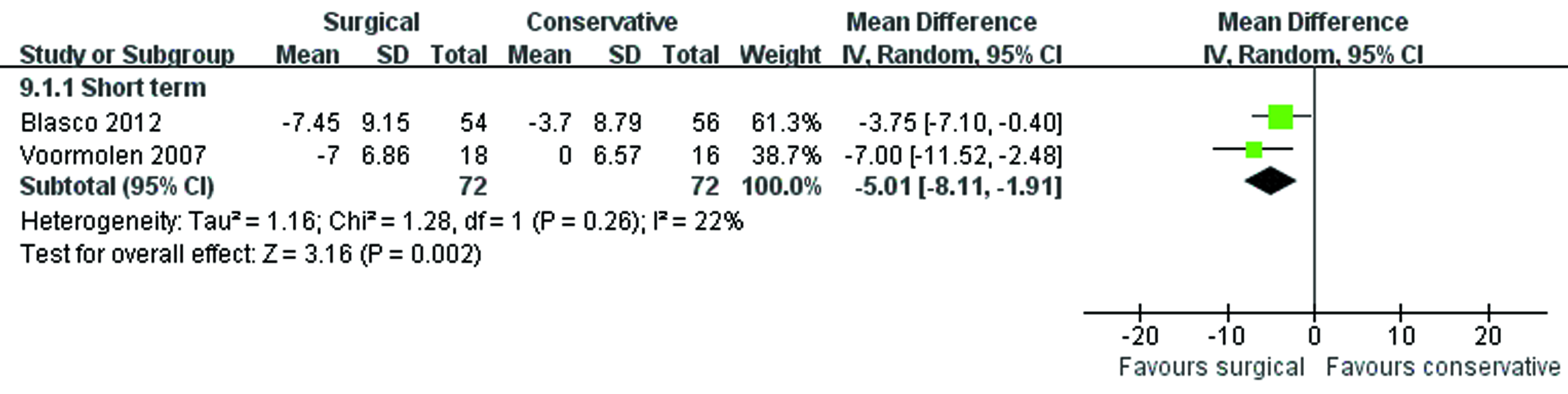

Supplement: S5 Fig — Short term: not longer than 3 months; Markers represent point estimates of mean difference, marker size represents study weight in random-effects meta-analysis. Horizontal bars indicate 95% confidence intervals. CI, confidence interval; IV, inverse variance. (TIF) [file pone.0127145.s007.tif]

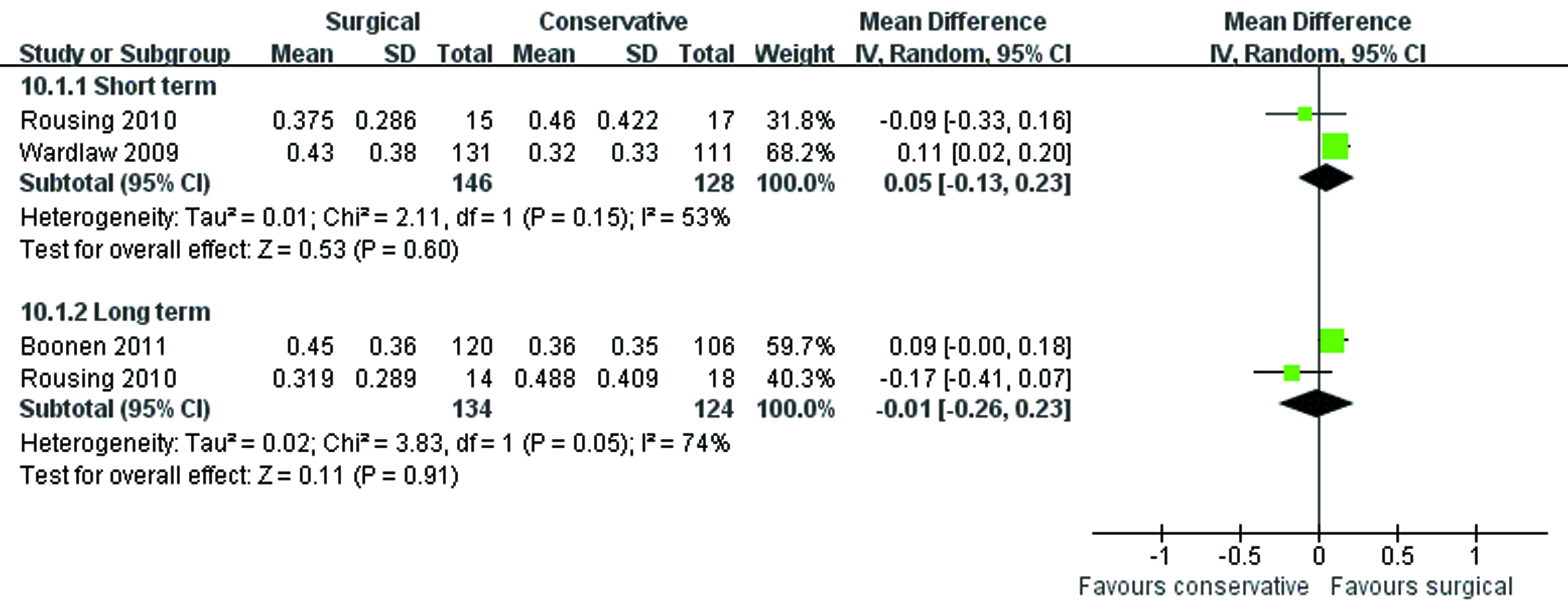

Supplement: S6 Fig — Short term: not longer than 3 months; Long term: 12 months or more; Markers represent point estimates of mean difference, marker size represents study weight in random-effects meta-analysis. Horizontal bars indicate 95% confidence intervals. CI, confidence interval; IV, inverse variance. (TIF) [file pone.0127145.s008.tif]

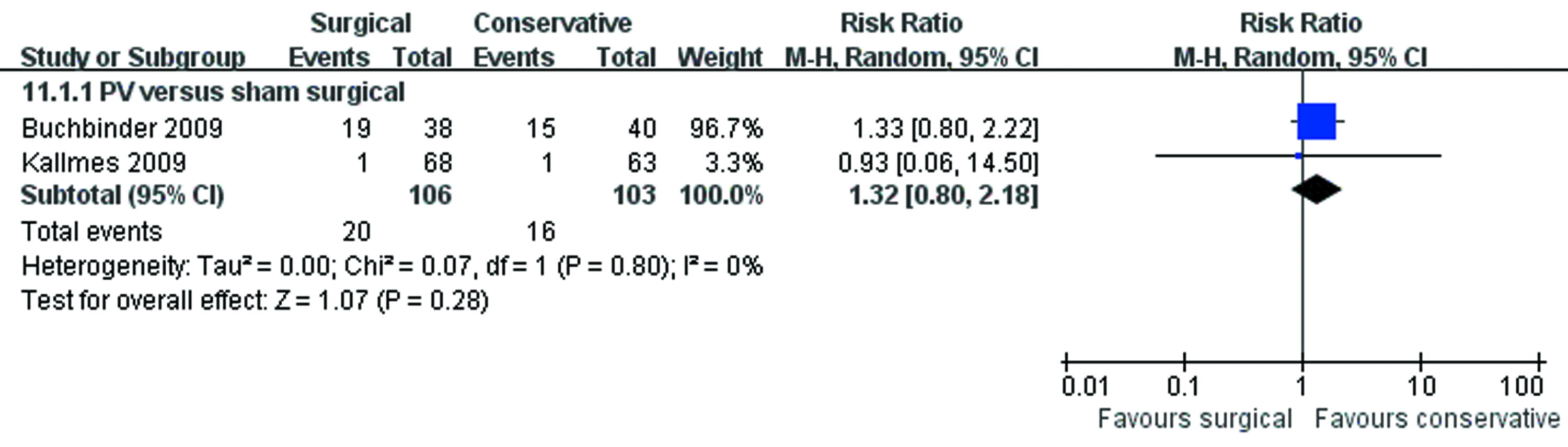

Supplement: S7 Fig — Markers represent point estimates of risk ratios, marker size represents study weight in random-effects meta-analysis. Horizontal bars indicate 95% confidence intervals. CI, confidence interval; M-H, mantel-haenszel. (TIF) [file pone.0127145.s009.tif]

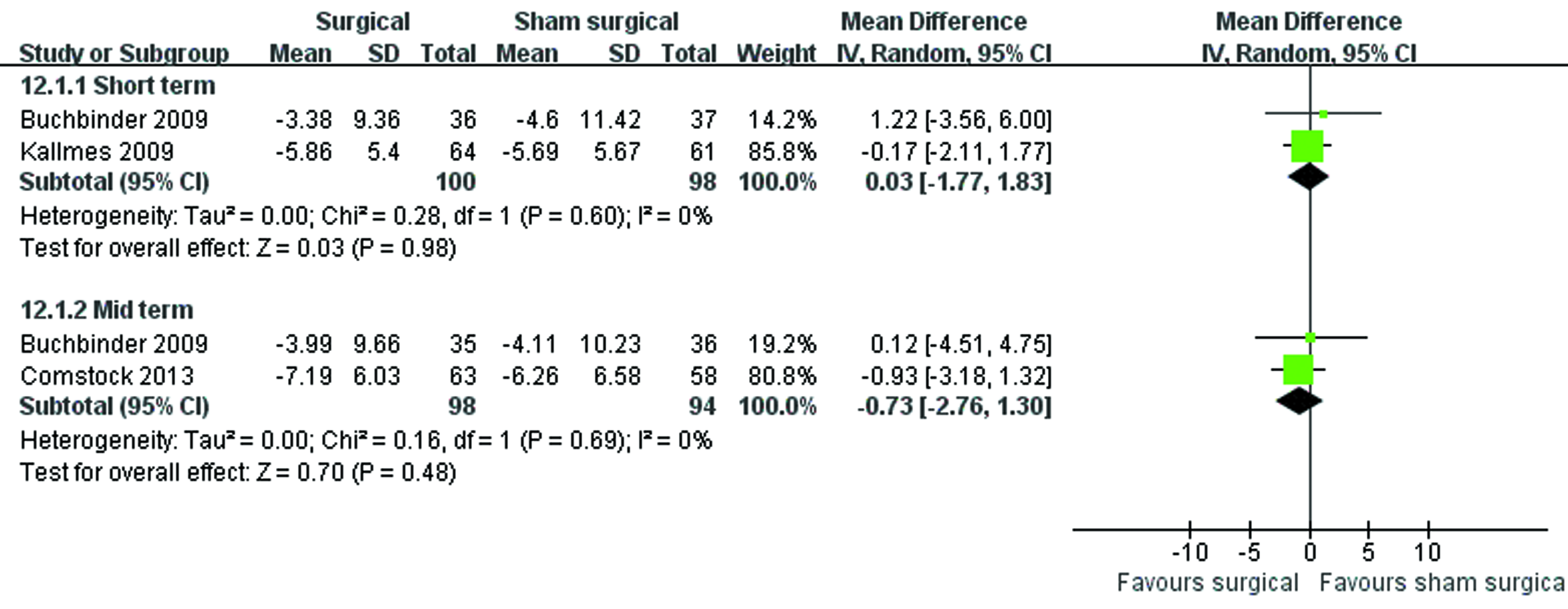

Supplement: S8 Fig — Short term: not longer than 3 months; Mid term: 6 months; Markers represent point estimates of mean difference, marker size represents study weight in random-effects meta-analysis. Horizontal bars indicate 95% confidence intervals. CI, confidence interval; IV, inverse variance. (TIF) [file pone.0127145.s010.tif]
